# Academic program recommendations for graduate degrees in medical physics: AAPM Report No. 365 (Revision of Report No. 197)

**DOI:** 10.1002/acm2.13792

**Published:** 2022-10-08

**Authors:** Jay W. Burmeister, Nathan C. Busse, Ashley J. Cetnar, Rebecca R. Howell, Robert Jeraj, A. Kyle Jones, Steven H. King, Kenneth L. Matthews, Victor J. Montemayor, Wayne Newhauser, Anna E. Rodrigues, Ehsan Samei, Timothy V. Turkington, Mary P. Gronberg, Brian Loughery, Alison R. Roth, Michael C. Joiner, Edward F. Jackson, Paul A. Naine, Leonard H. Kim

**Affiliations:** ^1^ Department of Oncology Detroit Michigan USA; ^2^ Colorado Associates in Medical Physics Colorado Springs Colorado USA; ^3^ Department of Radiation Oncology The Ohio State University Columbus Ohio USA; ^4^ Department of Radiation Physics Houston Texas USA; ^5^ Department of Medical Physics Madison Wisconsin USA; ^6^ Penn State Health Milton S. Hershey Medical Center Hershey Pennsylvania USA; ^7^ Department of Physics and Astronomy Baton Rouge Louisiana USA; ^8^ Germantown Academy Fort Washington Pennsylvania USA; ^9^ Mary Bird Perkins Cancer Center Louisiana State University Baton Rouge Louisiana USA; ^10^ Department of Radiation Oncology Duke University Durham North Carolina USA; ^11^ William Beaumont Hospital Dearborn Michigan USA; ^12^ Barrow Neurological Institute Phoenix Arizona USA; ^13^ Department of Oncology Wayne State University Detroit Michigan USA; ^14^ Elekta, Inc. Crawley UK; ^15^ Department of Radiation Oncology MD Anderson Cancer Center at Cooper/Cooper Medical School at Rowan University Camden New Jersey USA

**Keywords:** Medical Physics Graduate Education

## 1 PRELIMINARY DISCUSSION

This report details curricular recommendations for graduate degrees in medical physics and serves as an update to Report No. 197. In this section, we review the history of American Association of Physicists in Medicine (AAPM) curricular recommendations, present the aims of this report, and detail how these recommendations should be interpreted.

The first AAPM publication on curricular recommendations for graduate education in medical physics was AAPM Report No. 44, published in 1993, describing the recommendations for the Master of Science Degree in Medical Physics.^1^ AAPM Report No. 79 was published in 2002 and established a core curriculum for all graduate training in medical physics, as well as more specific education and training associated with the individual subspecialties in medical physics.^2^ In 2009, Report No. 79 was updated and published as AAPM Report No. 197, and in 2011, AAPM Report No. 197S was published on the essential didactic elements for alternative pathway entrants into the clinical medical physics profession.^3^, ^4^ Report No. 197S defined the curriculum for a postdoctoral certificate program in medical physics, the first of which was accredited by Commission on Accreditation of Medical Physics Education Programs (CAMPEP) in 2011. The AAPM Working Group on the Revision of Report No. 44 was initially created with the charge of periodically reviewing and updating the recommended curriculum for medical physics graduate education programs. In 2012, the AAPM renamed this as the Working Group on Medical Physics Graduate Education Program Curriculum (WGMPGEPC). The WGMPGEPC was further charged to ensure that the graduate education curriculum reflects current needs of clinical practice and provides a broad foundation upon which to base future innovations. The profession of medical physics has undergone substantial growth and change over the years since the publication of Report No. 197 in 2009, and the WGMPGEPC has updated the curricular recommendations to reflect these changes and prepare the profession for the future.

There exists a common core of similar coursework across all programs accredited by the Commission on Accreditation of Medical Physics Education Programs, Inc. (CAMPEP), yet each program has the latitude to adapt its curriculum to best leverage the strengths and resources of its faculty and host institution. The number of CAMPEP‐accredited graduate programs has more than doubled since the publication of Report No. 197, increasing from 24 in 2009 to over 50 in 2021. A common goal of the AAPM curriculum recommendations is to ensure that the core curriculum meets the current and anticipated future needs of the medical physics profession. The curriculum recommendations in this report support these aims in three ways. They provide guidance to graduate programs in medical physics regarding the topics that should be covered in their curricula. They provide guidance to instructors regarding the breadth of coverage of relevant topics. Finally, they provide a basis for developing standards for graduate medical physics education. The curriculum provides substantial detail within the topics provided for each section. This is primarily to assist graduate programs and course instructors, and it is not our intention to recommend that any accrediting or supervisory body would expect that a program would cover every item explicitly mentioned in the curriculum. A bibliography of suggested resources, categorized by topical area, is included, and entries are duplicated, as appropriate, when relevant to multiple topical areas.

There is a considerable degree of intellectual diversity of students entering graduate programs, including previous degree(s) earned, courses taken, and topics previously learned. The recommendations on graduate medical physics curricula in this report are designed to apply to all students. However, individual programs should determine whether to give credit to incoming students with previous course work that fulfills didactic medical physics training requirements (e.g., previous study of anatomy and physiology).

One major change since the publication of Report No. 197 is the requirement to complete an accredited residency training program to gain knowledge and skills needed to practice independently as a qualified medical physicist and to acquire professional board certification. The residency increases the amount of practical clinical training by at least 2 years, yet there remains a substantial benefit to providing practical learning experiences during graduate education. Benefits of this include baseline learning for all students (including those who pursue nonclinical careers, e.g., in industry and government), reinforcement of didactic learning, enhanced preparedness for clinical research, and preparedness to enter residency training. It is important for programs to design curricula to strike an appropriate balance between theoretical coursework and practical experiences. Graduate programs should provide ample opportunities for practical, hands‐on experiential learning in the clinical and laboratory environments. The practical learning experiences appropriate to graduate education are narrower in scope, more selective in topics, and more limited in time when compared with those of a residency program. Although many practical clinical topics are explicitly mentioned in this curriculum, it is left to the individual program to determine the breadth and depth of coverage.

The graduate certificate program remains an important part of the medical physics training infrastructure as the didactic medical physics preparation for alternative pathway entrants into medical physics practice. AAPM Report No. 197S defined the essential elements of this didactic preparation. As Report No. 365 supersedes Report No. 197, it also provides guidance to supersede the supplemental Report No. 197S. The topics defined here as Sections 2.1.1–2.1.10 represent the core elements identified during the development of this report. Topics in Sections 2.1.1–2.1.6 match those identified as essential didactic elements in Report No. 197S; however, their content has been updated. Topics in Sections 2.1.7–2.1.9 (“mathematical and statistical methods”, “computational methods and medical informatics”, and “research methods”) are the ones that may have been covered in the prior training of individuals entering the profession through the alternative pathway. Situations requiring remediation may or may not require the completion of full courses. This leaves topic in Section 2.1.10, “professionalism (leadership, ethics, communication),” as the notable addition to the recommendations of Report No. 197S. Report No. 197S recommended a curriculum, including a minimum of 18 credit hours of didactic coursework. Report No. 365 recommends the minimum curricular recommendations from Report No. 197S plus the delivery of training in professionalism. This may increase the credit hour requirements for programs that deliver this training within a formal didactic course. The AAPM recently commissioned a task group on Alternative Pathway Candidate Education and Training (TG‐298) to provide updated recommendations for the alternative pathway.^5^ Although Report No. 365 aims to provide overarching curricular recommendations for all graduate education programs in medical physics, TG‐298 aims to provide recommendations on the education and training of alternative pathway entrants into the medical physics profession. One important recommendation from TG‐298 is that programs provide clinical experience and exposure to the application of the didactic material. This recommendation provides support for the emphasis of practical clinical training within graduate education programs. A related recommendation by TG‐298 is that online delivery of curricular material should be carefully evaluated to assure that it does not limit the student's exposure to experiential, clinical aspects of medical physics. Lastly, TG‐298 recommends that programs include ethics and professionalism as a component of their core curriculum. We have included those components in the recommendations in this report.

The first professional doctorate degree in medical physics (DMP) was created in 2009 and accredited by CAMPEP in 2010. The DMP includes the same core curricular elements as the MS and PhD in medical physics and therefore does not warrant substantial changes in the curricular recommendations for graduate medical physics education. The DMP does provide additional opportunities and incentives for the inclusion of elective coursework that may be valuable for clinical practice, such as business and management coursework. These additional courses and others may also be valuable for students not intending to pursue clinical careers. Finally, DMP curricula must include clinical training of sufficient depth and breadth to prepare the student to become a qualified medical physicist. This training is the purview of other reports such as AAPM Report No. 249^6^ and Report No. 373^7^ and is not within the scope of this report.

An aim of the recommendations on graduate curricula presented in this report is to identify the salient topical areas of medical physics graduate education required to prepare trainees for current and future practice in this profession. As such, current technology, techniques, and methodology are often explicitly identified. It is, however, often instructive for trainees to understand historical aspects of medical physics technology and practice in order to understand the evolution of our practice and help guide its future. Such historical context is not always explicitly mentioned within this curriculum. However, it is assumed to be incorporated within medical physics graduate education where useful for the benefit of our trainees.

Similarly, another aim of this report is to prepare students and programs to adapt to future changes in the scope and nature of medical physics applications. This report represents a snapshot of current education and training requirements; however, we must also equip students with the education and skills necessary to contribute to, and be leaders of, future advances in medicine. Future contributions of physicists to medicine are often driven by understanding and training outside of traditional core topics. As such, training in nontraditional areas facilitates potential future contributions to science and medicine in general, and this emphasis has been explicitly incorporated into this curriculum. We should aspire to train our students to be multidisciplinary experts who are leaders in the science of medicine, who ensure the highest quality care and safety, and who initiate and create changes that enhance patient care. Incorporating these aspects and others that will arise in the future requires an increase in the breadth of education and training in medical physics. Programs should minimally strive to provide familiarity in these nontraditional areas, as it would be impossible to provide mastery in them all. Instilling critical thinking and lifelong learning skills will allow medical physicists to continue to enhance their ability to contribute to the science of medicine. Many nontraditional topics as well as applications of didactic material may be provided within seminar and/or practical application courses. Graduate programs are not expected to develop specific expertise in all of these areas, but outside lecturers and material developed by other departments and/or programs may be leveraged to fill these gaps.

The recognition that graduates from medical physics graduate programs may choose to enter nonclinical careers has encouraged the creation and promotion of didactic elements of graduate education aimed at best preparing those who enter nonclinical careers. The AAPM created the Working Group to Promote Non‐Clinical Career Paths for Medical Physicists in 2016, and the Working Group for Non‐Clinical Professionals in 2018. In order to address the educational needs of some nonclinical careers, in particular industrial career paths, we have included education and training in “Industry and Regulatory” as a component of the curricular recommendations in this report. It should be emphasized, however, that these training elements would also benefit those medical physicists that choose clinical career paths, as many of the challenges (e.g., interaction with industry, good business practices) are areas of essential strength of clinical medical physicists as well.

The provision of training in research is an important element in preparing for the future of our profession. Indeed, the AAPM description of the role of the medical physicist states that “medical physicists play a vital and often leading role on the medical research team.” This includes both basic and clinical research and the problem‐solving skills of the medical physicist. As such, we must provide the medical physics student with more than knowledge. We must provide the understanding that allows them to think beyond the present, to do more than just prescriptive problem‐solving, and to innovate and solve previously unsolved problems. Additionally, medical physicists have not been sufficiently integrated into clinical trials research in the past, particularly in leadership roles, which has often negatively impacted the quality of clinical research. Specific recommendations for such training are provided in this report that addresses particular areas of research training. In addition, every effort should be made to foster critical thinking skills throughout the education and training of future entrants into our profession, as this is a distinguishing feature of a medical physicist and one that makes us valuable to the medical profession.

Finally, this report is primarily intended to provide recommendations on *what* to teach rather than *how* to teach it. The recommended depth of understanding for each topic is not specifically prescribed here, and it is left to the individual program to determine, for each recommended topic, whether mere familiarity is sufficient or whether deep understanding and mastery of a topic is warranted. The application of Bloom's taxonomy or other models to classify educational learning objectives is helpful in determining specific goals for competence.^8^ We also do not recommend specific courses but rather curricular content presented within topical areas. As there can be significant overlap between topical areas, we have attempted to simplify the report by assigning particular curricular elements to only one topical area and referencing them to that area from others in which they may be relevant. Finally, we make no recommendations on pedagogical aspects of graduate education, including how or when material is provided. Discussion of the merits or application of online coursework, flipped classrooms, or other aspects related to the cognitive science involved in developing and delivering education is not included here.

Section 2 provides a description for each of the sections within the report, whereas Section 3 provides detailed curricular recommendations where applicable. Sections 2.1 and 3.1 provide recommended core topics, whereas Sections 2.2 and 3.2 provide additional (optional) topics. Sections 2.3–2.7 and 3.3–3.7 represent professional specializations (diagnostic imaging, nuclear medicine, radiation therapy [RT], medical health physics, and industry). These respective sections provide curricular recommendations for students specializing in each of these professional practice areas. These areas are denoted as optional and some programs do not provide an option for specialization.

DATA AVAILABILITY STATEMENT

Data sharing is not applicable to this article as no new data were created or analyzed in this study.

## 2 TOPICAL DISCUSSION

### 2.1 Core topics

#### 2.1.1 Radiological physics and dosimetry

An understanding of the structure of matter and the manner in which ionizing radiation interacts with it is critical to the application of radiation to imaging, nuclear medicine (NM), RT, and health physics. This material builds off concepts of modern physics and is recommended within an introductory course to serve as a foundation for many of the other sections contained within this curriculum. The primary learning objectives include an understanding of individual interaction mechanisms, including both the physics involved in describing the probability for each interaction and the way in which energy is dissipated in the interaction. The student should be able to apply these concepts to all particles of interest, including uncharged particles (photons and neutrons) and charged particles (electrons, protons, alpha particles, etc.) with the ability to analyze differences between the mechanisms involved in charged and uncharged particle interactions. The physics and mathematics of radioactive decay should be understood along with all decay mechanisms. A broad understanding of the measurement of radiation, specifically including the measurement of absorbed dose, should be attained, including radiation dosimetry concepts, techniques, and equipment. This should include concepts such as radiation equilibria, cavity theory, microdosimetry, and all quantities and relationships involved. Finally, the student should be familiar with the various types of radiation dosimetry equipment, along with their mechanisms of operation and limitations.

#### 2.1.2 Radiation protection and radiation safety

Radiation protection and safety pervades the various subspecialties of medical physics. Although technologies will change, having a fundamental understanding of how radiation works and how to protect oneself and others are crucial principles to the medical physics profession. A comprehensive study of radiation protection and safety could be structured by providing the answers to these major questions: Why does radiation need to be managed? What can you do to manage radiation exposure? How can you detect radiation? How much exposure can you safely receive? For whom is this important? How can you develop a safety culture? By posing these questions, a broad spectrum of topics can be discussed, including fundamental physics interactions, biological effects of radiation, and basic principles of radiation protection. Special attention is given to protection and safety of the radiation worker, patients, the public, and the environment. It is important to consider the present regulatory environment and the interactions with the recommendations from multiple organizations outside of the AAPM. Complementary tutorial instruction could include a sequence of laboratory experiences focusing upon radiation detection instrumentation, shielding methodology, and clinical applications for radiation protection and safety. The emphasis in this topic is to provide a broad knowledge base of radiation safety and protection supportive of the varied environments of medical physics practice.

#### 2.1.3 Fundamentals of imaging in medicine

Medical imaging is a foundational component of medical physics and has been developed and advanced over decades to become a cornerstone of healthcare. Because of the ubiquitous use of imaging, all medical physicists need a working knowledge of key imaging physics concepts. The core competencies presented in this section include concepts of image processing, image display and image quality; image reconstruction from projections; and the key hardware, software, and operational details of each imaging modality. These modalities are projection X‐ray imaging (radiography, mammography, and fluoroscopy), volumetric X‐ray imaging (computed tomography [CT], cone‐beam CT, and tomosynthesis), nuclear imaging (scintigraphy, single‐photon emission CT, and positron emission tomography [PET]), ultrasound imaging (echo 2D and 3D imaging, and Doppler imaging), and magnetic resonance imaging (MRI). The details listed for each core competency indicate the minimum depth of coverage. The core competencies presented in this section can be supplemented by application‐specific knowledge about targeted use of imaging technologies, whether for imaging or therapeutic applications.

#### 2.1.4 Fundamentals of radiation therapy physics

RT is a foundational component of medical physics, with 2/3 of all cancer patients receiving RT and numerous applications for RT for nonmalignant conditions. Medical physicists in all disciplines should have basic familiarity with the clinical, technological, and radiobiological concepts involved in RT. The significant overlap of imaging and NM with RT and the associated potential for collaborative work across these specialties underscores the need for cross‐disciplinary training. The core elements of RT are presented here, including clinical and radiobiological principles, equipment and technology used for RT, specific treatment techniques and principles of radiation protection and quality management. The information presented here should ideally be supplemented by practical exposure to these technologies and techniques in the clinical environment.

#### 2.1.5 Radiobiology

All subspecialties of medical physics require an understanding of the biological effects of radiation. Specifically, radiobiological principles comprise foundational knowledge in underpinning theories of radiation protection, RT, radiation imaging of humans, and NM. Radiobiology provides the basic connection between microscopic and molecular interactions of radiation with cellular and tissue responses. This material provides a solid biological and physiological background for understanding the effects of radiation on human tissues and cancers and the resulting safety policies and therapy regimens. These topics should be presented in a cohesive and consistent manner, not distributed among subspecialty applications such as RT physics, imaging physics, radiation protection and safety, and NM.

#### 2.1.6 Anatomy and physiology

Anatomy and physiology underpin the entirety of medical physics. Familiarity with normal anatomy is fundamental to radiotherapy treatment planning and medical imaging optimization. Cancer biology must also be understood at a basic level, as it drives many aspects of RT and medical imaging. Key linking concepts can be covered where they exist, such as cardiotoxicity from radiotherapy and the link between normal glucose metabolism and PET imaging. An organ system approach is logical for presenting the content in this section, with particular emphasis on normal imaging appearance, common imaging tasks related to pathology, and disease sites related to radiotherapy.

#### 2.1.7 Mathematical and statistical methods

Competency in mathematical methods is foundational to the understanding of medical imaging, radiological physics, and dosimetry. Incoming graduate students should have a strong background in mathematics demonstrated by their undergraduate or graduate coursework. Medical physics graduate training should enhance the understanding of mathematical techniques as they relate to medical imaging, computational science, and optimization. Graduate training should also provide a foundation in statistical methods as it relates to experimental design and analysis.

#### 2.1.8 Computational methods and medical informatics

It is becoming increasingly important that medical physicists possess a working knowledge of computational methods and informatics. Graduate curricula should include the basics of programming and machine learning as they relate to the many potential applications in medical physics. They should also include informatics as it relates to medical image storage and transfer. These skills could be developed through opportunities to practice implementation such as class projects, assignments, and research.

#### 2.1.9 Research methods

Students should be exposed to and participate in research and be familiar with research methods, ethics, and scientific communication, which includes academic writing, reviewing, and presentation. In addition, protocol and grant writing, clinical translation/implementation, literature search and reading, and laboratory management are important skills with which students should become familiar. As the experience a student gains from their research endeavors will vary depending on individual advisors and projects, programs should consider how to ensure the consistency of development of research skills across all students. The delivery of a seminar series, such as journal clubs or presentations from students, faculty, and invited speakers is one useful mechanism for the development of research skills. Although all formats should expose students to a breadth of current research topics, different formats inevitably emphasize different skills such as literature review, communication and scientific presentation skills, and others. Programs should keep this in mind when designing the seminar format to give students the opportunity to develop all of these skills. Programs that do not require a thesis project or a seminar series should consider how their students will develop the skills traditionally associated with such offerings. Alternatives practiced by some programs include class projects, laboratory sections, “special topics” courses focused on current literature and research, and student attendance at scientific conferences. Finally, exposure to areas such as clinical trials, grant/protocol writing, laboratory management, and clinical translation/implementation may be best acquired via a faculty advisor/mentor and is perhaps more suitable in a PhD program. As the certificate program pathway is open only to individuals holding a PhD degree, it is anticipated that these research requirements would have been fulfilled prior to entry into the certificate program.

#### 2.1.10 Professionalism (leadership, ethics, and communication)

The medical physicist will be routinely involved in interactions with professional colleagues, collaborating clinicians, trainees, patients, research subjects, administrators, and/or support staff, to name just a few. In all such interactions, a number of critical skills must be applied. First and foremost, the medical physicist must understand the ethical obligations and responsibilities of this role. As many medical physicists will be involved in leadership and management within the hospital and university setting, as well as serving as the de facto leader of the technical, quality, and safety aspects of a clinical department, the development of leadership skills is very important. These skills allow the medical physicist to have an appropriate and sufficient influence within these areas, for example, to influence the safety culture of a clinical department. Good communication skills are critical for leadership as well as for patient and clinician interactions. Such skills not only enhance leadership capabilities but also efficiency and accuracy in clinical collaboration as well as facilitating the most effective patient care.

### 2.2 Additional topics (optional)

#### 2.2.1 Biology/oncology/medicine

Radiation biology and anatomy and physiology are essential for medical physics competency. However, additional training in biology subfields can provide further competency both for research and in clinical practice, enhancing the ability of medical physicists to contribute to medical science and practice in general. Some example areas include biochemistry and biomolecules, cardiology, computational biology, epidemiology, genomics, immunology, neurobiology, oncology, pathology, and clinical pharmacology. Although it would be impossible to include all of these topics within a graduate program in medical physics, familiarity with these topics, along with preparation in health science terminology, helps medical physicists better communicate with clinicians and other research scientists and contribute more integrally to clinical and research efforts. This additional training further facilitates the identification and exploration of nontraditional applications of medical physics and thus expands our contribution to medicine.

#### 2.2.2 Advanced physics, engineering, and computer science

Elective coursework provides students with the opportunity to broaden their understanding of related fields or deepen their knowledge of medical physics specialties. Advanced physics disciplines of interest to the medical physicist may include nuclear physics, electricity and magnetism, optics, and solid state physics. Engineering coursework can be found in the biomedical, nuclear, electrical and computer engineering disciplines. Computer science electives should focus on topics to prepare medical physicists to be effective collaborators within the scientific community. Example coursework may cover statistical software packages, team programming practices, including version control and best practices for readability, and artificial intelligence.

#### 2.2.3 Frontiers in medical physics and opportunities outside of medical physics

Frontiers in medical physics represent areas in which physics has begun to contribute to the improvement of medical care and/or has the potential to further improve human health. In RT, examples include FLASH, RT for non‐cancer treatments (e.g., cardiac ablation), radiomics, interplay with other therapies (e.g., immunotherapy, photodynamic therapy [PDT]). In imaging, examples include radiomics and theranostics, interferometry imaging, biophotonics, magnetoencephalography, and alternative (monochromatic) X‐ray sources. In NM, examples include novel radiotracer development for radiomics and theranostics. Additional opportunities outside medical physics represent areas outside the current landscape of medical physics practice. Areas for potential involvement include surgery (image guidance), pathology (image display, automation), ophthalmology (optical modeling), dentistry (3D modeling), orthopedics (motion analysis), cardiology (electrophysiology), neuroscience, and psychology. There is no expectation that any particular program cover these materials, rather this functions as a survey of potential topics. Incorporation of these topics in a specific program should reflect that program's strengths and research.

#### 2.2.4 Business applications in medical physics

Medical physics as a profession has multiple facets that often interact and overlap with business principles in a wide range of areas. Although it is not necessary to have an intricate knowledge of business aspects, it is important to be able to effectively communicate in these areas. As with all roles, the effectiveness of the function of medical physics is based on sound business decisions being made. A medical physicist may find use for these skills working in a clinic, working for a vendor, or working in a physics consulting company. In addition, any medical physicist functioning in a leadership role will eventually require business skills. A medical physicist's role will ultimately determine the level of knowledge that is required, whether it be large commercial operations or a single consultant. As such, the skills below are a broad cross section of areas that will provide a foundation for this knowledge, and the topics are intended as a baseline dependent on the demands of the individual's situation. Many of these business skills are strongly correlated with, and can be considered simply another facet of, skills outlined in other sections of this curriculum.

#### 2.2.5 Teaching for medical physicists

Effective teaching is an increasingly important skill for all medical physicists. Indeed, medical physicists are often asked to teach within medical physics, RT technology, dosimetry, and residency programs and to provide training in‐services for other clinicians. As such, it is valuable for medical physicists to have an understanding of adult learning theory and the different educational pedagogies that may be used. As teachers tend to teach in the manner in which they were taught, the most effective way to teach students about different methodologies along with the potential advantages and disadvantages of different pedagogies is not to lecture about them, but rather to have students experience those pedagogies firsthand in the classroom. Physics Education Research has clearly shown that didactic lecture, while being the most efficient for the lecturer, is likely the least effective method of teaching for the majority of physics students. The more active and hands‐on the pedagogy, the more engaging and effective the time spent in class. However, it has also been shown that alternate pedagogies can sometimes take significantly more time to cover the same amount of material as a traditional didactic lecture. This means that there is still value in a well‐prepared and well‐delivered lecture. The topics listed here provide methods designed to create the most effective learning environment for the student.

### 2.3 Diagnostic imaging specialization (optional)

Medical imaging is a foundational component of medical physics. Every medical physicist must master core competencies, including the principles of image formation, imaging hardware and software, and the optimization and constraining of the imaging process. However, those physicists specializing in diagnostic imaging (DI) require additional depth in imaging physics theory and practical applications. The topical outline here replicates and extends the outline provided in fundamentals of imaging. This enhanced knowledge includes the principal concepts for each DI modality: projection X‐ray imaging (radiography, mammography, and fluoroscopy), volumetric X‐ray imaging (CT, cone‐beam CT, and tomosynthesis), ultrasound imaging (echo and transmission 2D, 3D, and Doppler imaging), MRI (MRI methods and spectroscopy), and information technology (picture archive and communication system [PACS], displays, EMR, and safety and quality monitoring). The nuclear imaging specialization (planar, single‐photon emission CT, and PET) is described in a separate section, although there is substantial overlap in many topics. The training of a DI physicist must also incorporate experiential knowledge gained through hands‐on practical activities, as they pertain to optimal use of imaging technologies in the clinic, including quality assurance (QA), applications, and processes to customize a procedure to the patient. The clinical priority is to enable and ensure optimized, reliable, quantitative, and safe use of the imaging technologies.

### 2.4 Nuclear medicine specialization (optional)

The work of NM physicists spans radionuclide production, radiation protection, scanner operation (including calibration, maintenance, and QC), image reconstruction, and image analysis (including tracer kinetic modeling) and creating new hardware and algorithms for NM applications. Although most physicists’ jobs do not entail all of these components, it is important that basic NM physics education be broad because of both the different job opportunities that exist and their fundamental interrelatedness. NM inherently draws on the related contributions of physicists, chemists, pharmacists, physicians, technologists, and others. Some familiarity with the other professionals’ disciplines can be beneficial for the physicist as part of the interdisciplinary team and essential for understanding NM in general. NM physics education should cover the principles, devices, and algorithms used in the field. A broad background provided in medical physics core topics is essential. It is widely regarded that therapeutic applications of NM will continue to increase and that more patient‐specific dosimetry, specifically based on imaging/therapeutic compound pairs, will be warranted, providing a good opportunity for broadly trained NM physicists. Much NM physics (especially that related to the imaging devices) is performed by physicists practicing in DI. From an educational perspective, a physicist broadly engaged in NM, whether as a DI physicist or as a dedicated NM physicist (or even as an RT physicist), should have the in‐depth NM didactic background recommended in this section.

### 2.5 Radiation therapy specialization (optional)

RT is a foundational component of medical physics. As such, medical physicists in all disciplines should have basic familiarity with the clinical, technological, and radiobiological concepts involved in RT. This section provides both the historical background and fundamental bases for the therapeutic use of ionizing radiation, along with a detailed description of the equipment and techniques used in modern RT, including all aspects necessary for clinical practice in radiation oncology. The wide variety of radiation sources, the technology associated with production and delivery of this radiation, and the clinical characteristics and utility of the various modalities used for RT are presented. The information presented here should be supplemented by practical exposure to these technologies and techniques in the clinical environment.

### 2.6 Medical health physics specialization (optional)

Health physics is a distinct profession with its own training programs, curricula, and professional society (Health Physics Society). Health physics contains subtopics, however, that are germane to medical physicists. Content from radiation physics, detection, and dosimetry; radiation protection and safety; and radiation biology is especially fundamental to many medical health physics topics. Although that content is fundamental, medical health physics students will need the greater depth covered here.

### 2.7 Industry specialization (optional)

Roles in industry and regulatory agencies require a diverse set of skills and knowledge to be able to effectively collaborate with team members with backgrounds that may differ substantially from those in a clinical environment. This sector of potential employment is critical in translating research/academic concepts to a product that can be put into clinical use. Often there is some mystery surrounding roles in this sector due to the variety of opportunities. Additionally, some of the topics suggested may be unfamiliar to candidates who have specialized in science. It is suggested that knowledge levels in these areas are attained to the understanding level of Bloom's taxonomy as an introduction to industry. The proposed topic lists are not exclusive nor comprehensive, and the content within these topics will differ over time as the landscape in this field changes rapidly. In addition, it should be noted that some subject areas are US specific; however, they could be adapted for other countries. It is, however, strongly recommended that any candidate who is considering a role in this sector gain awareness in each of these topics. With that said, it is also important to note that there is extensive detail within each of these topics, some having their own full degree programs associated with them. The goal should always be to have enough peripheral knowledge to conduct daily workplace operations unassisted and understand when it is necessary to reach out to experts in the respective topic.

## 3 TOPICAL OUTLINE

### 3.1 Core topics

#### 3.1.1 Radiological physics and dosimetry

1. Review of atomic and nuclear structure
  - Atomic structure, electron shells, electron shell filling, electron binding energy, electron excitation, and models of the atom
  - Nuclear structure, nucleons, nucleon binding energy, nuclear excitation, nuclear stability, and models of the nucleus
  - Unstable structures, line of nuclear stability, radioactive decay and de‐excitation
2. Classification of radiations
  - Basic physical quantities and units used in radiation physics
  - Types and sources of directly and indirectly ionizing radiations
  - Description of ionizing radiation fields
3. Quantities and units used for describing radiation fields
  - Radiant and rest‐mass energies
  - Fluence and fluence rate
  - Energy fluence and energy fluence rate
  - Monoenergetic and polyenergetic spectra
4. Quantities and units used for describing the interaction of ionizing radiation with matter
  - Interaction cross sections
  - Microscopic and macroscopic cross sections and their relationships
  - Kerma, collisional kerma, radiative kerma, and converted energy per unit mass
  - Absorbed dose
  - Exposure/air kerma
  - Relationships between exposure, kerma, and absorbed dose
  - Equivalent dose, quality factor, and radiation weighting factor
5. Indirectly ionizing radiations
  - X‐ray transitions, characteristic radiation, ionization versus excitation of atoms
  - Radiation from accelerated charge, production of bremsstrahlung, and Larmor relationship
  - X‐ray targets, bremsstrahlung yield
  - Photon beam quality and filtering
  - Energy deposition in matter by photon beams
  - Neutron sources and spectra
  - Neutron beam energy regimes
6. Interaction of indirectly ionizing radiation beams
  - Simple exponential attenuation
  - Half‐value layer, 10th‐value layer, attenuation coefficients, and interaction cross sections
  - Narrow versus broad beam attenuation and geometry
  - Buildup factor
  - Spectral effects in attenuation, beam hardening, and softening
  - Reciprocity theorem
  - Energy transfer coefficient, energy absorption coefficient
7. Photon interactions with matter
  - Thomson scattering/Rayleigh scattering
  - Photoelectric effect
  - Compton scattering
  - Pair production and triplet production
  - Photonuclear reactions
  - Relative predominance of individual effects as a function of energy and atomic number
  - Contributions of individual effects to the attenuation coefficient, energy transfer, coefficient, and energy absorption coefficient
8. Neutron interactions with matter
  - Neutron interactions, including scatter, absorption kinematics, and cross sections
  - Shielding consideration for neutrons
  - Neutron kerma and absorbed dose calculations in different media
  - Gamma‐neutron mixed field dosimetry
  - Neutron quality factor and radiation weighting factor
9. Charged‐particle interactions with matter
  - Stopping power (collisional and radiative), scattering power
  - Stopping power (electronic and nuclear)
  - Restricted stopping power, linear energy transfer (LET)
  - Continuous slowing down approximation and straight‐ahead approximation
  - Pathlength, range, projected range, energy, and range straggling
  - Charged particle transport (water‐equivalent thickness)
  - Energy distribution of charged particles (e.g., electrons and protons)
  - Calculation of absorbed dose from charged particles
  - Types of charged particle beams used clinically
10. Radioactive decay
  - Total and partial decay constants
  - Units of activity
  - Mean‐ and half‐life
  - Radioactive disintegration processes
  - Fluorescence yield, Auger effect
  - Parent–daughter relationships
  - Transient and secular equilibrium
  - Harvesting of daughter products
  - Radioactivation by nuclear interactions
  - Exposure rate constant and air‐kerma rate constant
11. Charged particle and radiation equilibria
  - Radiation equilibrium, “buildup”
  - Charged‐particle equilibrium (CPE), transient CPE, and conditions causing CPE failure
  - Relationships between absorbed dose, kerma, and exposure under CPE
12. Radiation detection and dosimetry
  - Principles of radiation detection/radiation detection mechanisms
  - Types and general characteristics of detectors and dosimeters
  - ICRU (International Commission on Radiation Units and Measurements) definitions of dosimetry quantities and units
  - Absolute versus relative dosimetry techniques
  - Interpretation of dosimeter measurements
  - Uncertainties and uncertainty budgets
  - Primary and secondary calibration standards and the chains of calibrations
13. Cavity theory
  - Bragg‐Gray cavity theory and corollaries
  - Spencer–Attix and Burlin cavity theories, Fano's theorem
  - Applications and limitations of cavity theory
  - Calculation of the mean stopping power using the method of moments of the energy loss distributions
  - Dose near interfaces
14. Ionization chambers
  - Basic characteristics of ionization chambers
  - Standard free air ionization chamber
  - Cavity (thimble) ionization chamber
  - Plane parallel chamber/extrapolation chamber
  - Ion chamber survey meters
  - Measurement of chamber current (differential mode) and charge (integral mode) and operation of electrometer
  - Mean energy required to create an ion pair
  - Saturation characteristics of ionization chambers: initial and general recombination
  - Correction factors applied to ionization chamber measurement (e.g., following the nomenclature and terminology of International Atomic Energy Agency [IAEA] TRS 398)
15. Calibration of radiation beams with ionization chambers
  - Cavity chamber calibration: air‐kerma in air and dose in water
  - Dosimetry protocols (e.g., AAPM TG‐51; IAEA TRS‐398)
  - Phantom materials for photon, proton, electron, and neutron beams
16. Radiation detectors and measurement techniques (principles of operation, detector geometry and response, calibration, and corrections)
  - Gas ionization detectors (ionization chamber, proportional counter, and Geiger‐Müller [GM] counter)
  - Scintillators and photosensors
  - Semiconductors
  - Film (radiographic film and radiochromic film and their use for relative and absolute dosimetry)
  - Thermoluminescent dosimeters, including excitation and de‐excitation of crystalline solids
  - Optically stimulated luminescence dosimeters
  - Calorimeters
  - Chemical (Fricke) dosimeters
  - Neutron detectors/gamma–neutron mixed field dosimeters
  - Miscellaneous detectors, for example, MOSFET (metal oxide semiconductors—field effect transistor), diamond detectors, three‐dimensional gels
17. Microdosimetry
  - Lineal energy, specific energy
  - Experimental microdosimetry and microdosimetric spectra
  - Relationship between LET and relative biological effectiveness (RBE)

#### 3.1.2 Radiation protection and radiation safety

1. Management of radiation risk
  - Rationale for the management of radiation risk
  - Examples of management strategies for radiation risk
  - Examples of past radiation incidents and events
2. Biological effects of radiation
  - Observed radiation injury
  - Non‐stochastic and stochastic responses
  - Biological experimental data base of radiation injury
  - Biological Effects of Ionizing Radiation Reports
  - UNSCEAR (United Nations Scientific Committee on the Effects of Atomic Radiation) Reports
  - Fetal dose
  - Biological effects of nonionizing radiation
3. Principles of radiation safety and radiation protection practices
  - As low as reasonably achievable
  - Time, distance, and shielding
  - Handling of radioactive sources and materials
  - Radioactive source management and security
  - Additional practical applications of radiation safety principles
4. Protective apparel/PPE
  - Materials used for protective apparel (lead vs. composite)
  - Ancillary equipment (e.g., shields, glasses, and gloves)
  - Effectiveness and implementation of monitoring of personnel
5. Shielding design principles
  - Directly and indirectly ionizing radiation
  - Primary particle shielding
  - Secondary–tertiary particle shielding
  - Dependence of shielding design on energy and particle type
  - Buildup parameterization/Archer equation
  - Modeling radiation environment
  - Interlocks and access control
  - NCRP (National Council on Radiation Protection and Measurements) shielding recommendations and techniques
6. Shielding for types of facilities
  - Diagnostic facilities
  - NM
  - Linac vaults
  - Brachytherapy suites
  - Proton/heavy ion accelerators
7. Applications of radiation detection for radiation safety
  - Detection techniques and instrumentation for radiation safety (including well counter, dose calibrator, liquid scintillation counter)
  - Appropriate selection of radiation detection/measurement technique
  - Radionuclide identification techniques and equipment
8. Statistics of radiation detection
  - Statistical interpretation of instrument response
  - Stochastic and non‐stochastic error analysis
  - Minimum detectable activity
  - Propagation of errors
9. Quantifying radiation
  - Units, kerma and absorbed dose, and dose equivalent
  - Operational dosimetry
  - Recommendations of the national and international organizations
  - Quality factors
10. Radiation monitoring of personnel
  - Detection devices and techniques for personnel monitoring
  - Integral and active devices
  - Dynamic range and response sensitivities
  - Occupational limits for personnel and public
  - Effective dose‐equivalent calculations for personnel using protective apparel (EDE1 and EDE2)
  - Pregnant workers and fetal dose limits
11. Internal exposure
  - National and international organization recommendations
  - Medical internal radiation dose (MIRD) dosimetry
  - Monitoring and radiation control (including biological assay and dispersion in a working environment)
  - Allowed limit of intake and derived air (or water) concentrations
12. Regulations and Recommendations for Radiation Protection and Safety
  - Definitions of radiation safety regulations versus recommendations
  - National and International Recommendations and Regulations (10CFR19‐70; 49USDOT300‐399, 198; 219SFDA 278; 290SHA; 42USPHS; 40USEPA)
  - Transportation of radioactive materials (RAMs) (labeling and transportation index)
  - Environmental dispersion and waste disposal
  - Regulatory oversight (US Nuclear Regulatory Commission [NRC] vs. Agreement States)
  - Recommendations from NCRP, International Commission on Radiological Protection, American College of Radiology (ACR), The Joint Commission (TJC), Conference of Radiation Control Program Directors

#### 3.1.3 Fundamentals of imaging in medicine

1. Radiation physics and detection for imaging
2. Fundamentals of digital image processing
  - Linear systems
  - Discrete signal processing
  - Pixel‐based operations: window, leveling, subtraction, and thresholding
  - Convolution and spatial domain filtering for smoothing and enhancement
  - Fourier‐space filtering
  - Magnification, interpolation, deformation, and registration
  - Segmentation
  - Analysis (e.g., similarity, cross‐correlation, texture, and shape)
  - Compression; lossy and lossless methods
3. Image quality
  - Resolution/unsharpness/blur: point‐spread function (PSF), modulation transfer function (MTF), and related metrics
  - Contrast, including radiographic contrast
  - Noise, including noise power spectrum (NPS)
  - Signal‐to‐noise ratio (SNR), contrast‐to‐noise ratio (CNR), detective quantum efficiency (DQE), contrast detail, and other composite metrics
  - Detectability and task‐based observer performance (false/true positive/negative, sensitivity and specificity, predictive value, precision and recall, *f*‐score)
4. Fundamentals of image reconstruction
  - Relationship of Radon and Fourier transforms: the central slice theorem
  - Analytical reconstruction: backprojection; ramp filter, filtered backprojection, noise‐reduction filters; inverse Radon transform
  - Fundamentals of iterative reconstruction: advantages, limitations, and common algorithms
5. X‐ray production
  - History of X‐ray physics
  - Bremsstrahlung production and characteristic radiation
  - Hot‐cathode X‐ray tube design (Coolidge design)
  - High‐voltage generators
  - Impact of design and operating parameters
  - Requirements for specific applications (mammography, CT, and fluoroscopy)
6. X‐ray imaging detectors
  - Historical impact of radiographic film on modern detectors and techniques
  - Intensifying screens
  - Storage phosphor plates
  - X‐ray flat‐panel detectors
7. Projection X‐ray imaging
  - Radiography: geometry, hardware, techniques, QA, and safety
  - Mammography: geometry, hardware, techniques, QA, and safety
  - Fluoroscopy: geometry, hardware, techniques, QA, and safety
8. Volumetric X‐ray Imaging
  - CT: geometry, hardware, techniques, artifacts, QA, and safety
  - Cone‐beam CT: geometry, hardware, and artifacts
  - Tomosynthesis: geometry, hardware, and artifacts
9. Ultrasound imaging
  - Ultrasound physics: interactions and propagation
  - Transducers: physics, materials; design and operation
  - US systems: ancillary components, operation
  - Image acquisition
  - Doppler flow measurement and Doppler imaging
  - Image quality: resolution (axial, lateral, elevational), artifacts, and noise
  - Bioeffects
  - Survey of ultrasound safety, QA, and regulatory requirements
10. Magnetic resonance imaging
  - History of magnetic resonance physics
  - Core topics in physics (magnetism, magnetic moments of nuclei, and induction)
  - Physics of nuclear magnetic resonance
  - NMR pulse sequences: hardware, pulse sequences, and weighting
  - Contrast agents
  - MR spatial signal localization: gradients, timing diagrams, and *k*‐space filling
  - Common MR image acquisition modes: timing diagrams and features
  - Image quality: effects of acquisition parameters
  - Artifacts and their causes
  - Introduction to advanced MR techniques: spectroscopy, elastography, functional MR, diffusion‐weighted imaging, and angiography
  - MR bioeffects
  - Principles of MR safety: personnel and patient safety
  - Siting and facility safety
  - QA and regulatory requirements
11. NM imaging
  - History: Becquerel, the Curies; de Hevesy; discovery of Tc‐99m
  - Detectors used in NM: scintillators, semiconductors, and gas‐filled
  - Counting (Poisson) statistics
  - Gamma‐ray spectroscopy
  - Radioactivity
  - Radionuclide production
  - Radiopharmaceuticals
  - Non‐imaging detectors in NM: dose calibrator, well counter, and thyroid uptake probe
  - Gamma cameras and scintigraphy
  - Single‐photon emission computed tomography (SPECT)
  - PET
  - Image reconstruction: importance of iterative reconstruction in NM
12. Radiation protection in imaging and NM
  - Entrance air kerma, entrance exposure; relationship to dose and dose equivalent
  - Radiation dose and risk: typical values for imaging procedures (all X‐ray and NM modalities)
  - Dose reduction in imaging: “right‐sizing” of techniques
  - NM‐specific radiation protection, QA, and safety, including room and personnel surveys, and shielding design/evaluation
  - Patient as source; hazards to staff; release criteria

#### 3.1.4 Fundamentals of radiation therapy physics

1. Overview of clinical oncology
  - Cancer incidence/etiology
  - Cancer classification/staging
  - Overview of oncology treatment modalities
2. History, evolution, radiobiological principles, and practical applications of RT
  - History and evolution of RT
  - Radiobiological principles of RT
  - Common clinical RT applications
3. Physics and operation of radiation oncology equipment
  - Linear accelerators
  - Other external beam RT equipment
  - Brachytherapy equipment
  - Technology for imaging in RT
  - Computerized treatment planning systems
  - Ancillary RT physics equipment (patient immobilization, dosimetry, and QA equipment)
4. External beam RT
  - External beam RT modalities
  - Target definition, treatment intent, and dose prescription criteria
  - Prescribing, reporting, and evaluating RT treatment plans
  - Treatment simulation techniques
  - Treatment planning techniques
  - Treatment delivery and verification
5. Brachytherapy
  - Radioactive sources
  - Treatment planning and dose specification
  - Treatment delivery techniques
6. Special techniques in radiation oncology
  - Rationale for special techniques and required physics resources and requirements
  - Examples of special techniques (e.g., total body irradiation [TBI], total skin electron therapy, intraoperative radiotherapy, stereotactic radiosurgery [SRS], stereotactic body RT, and radionuclide therapy)
7. Imaging for RT
  - Imaging for treatment simulation
  - Multimodality imaging for treatment planning
  - Imaging for treatment guidance, motion management, and verification
8. Radiation protection and quality management in radiation oncology
  - Shielding for simulation and treatment rooms
  - NRC and state regulations
  - Radiation protection programs

#### 3.1.5 Radiobiology

1. Radiation injury to DNA
  - Radiation chemistry of water
  - Structure of DNA and types of radiation‐induced lesions
  - Double‐strand breaks
  - Radiation dosimetry, microdosimetry, and ionization density and their relationship to DNA damage
2. Repair of DNA damage
  - Single‐strand repair pathways
  - Repair of double‐strand breaks
3. Radiation‐induced chromosome damage and repair
4. Chromosome biology and aberrations
5. Linear‐quadratic model
6. Survival curve theory
  - Cellular sensitivity
  - Mechanisms of cell killing
  - Target theory
  - Survival curve models (single‐hit multi‐target, linear quadratic)
7. Concepts of cell death
  - Reproductive cell death
  - Programmed cell death
8. Cellular recovery processes
  - Types of radiation damage
  - Potentially lethal and sublethal damage
  - Fractionation effect
  - Dose rate effects
9. Cell cycle
  - Cell kinetics and cycle phases
  - Radiosensitivity and cell cycle position
  - Radiation effects on cell cycle
10. Modifiers of radiation response: sensitizers and protectors
  - Oxygen effect and other radiosensitizers
  - Radioprotection
11. RBE, oxygen enhancement ratio (OER), and LET
  - LET
  - RBE
  - OER
12. Cell kinetics
  - Cell cycle and quantitation of its constituent parts
  - Growth fraction and cell loss from tumors
  - Autoradiography and flow cytometry
  - Growth kinetics of human tumors
13. Radiation injury to tissues
  - Tissue and organ anatomy
  - Biologic endpoints, expression, and measurement of damage
14. Temporal aspects of radiation effects—acute and late effects
  - Acute and late responding normal tissues
  - Pathogenesis of acute and late effects
  - Different kinds of late responses
  - Residual damage/radiation syndromes
15. Histopathology
  - General morphology of radiation injury
  - Morphology of cell death
  - Morphologic changes in irradiated tumors
16. Tumor radiobiology
  - Basic tumor structure and physiology
  - Importance of hypoxic cells in tumors and importance of reoxygenation
17. Time, dose, and fractionation
  - The 4 R's of radiobiology
  - Volume effects
  - The basis of fractionation
  - Dose–response relationships for early and late responding normal tissues
  - Hyperfractionation and accelerated treatments
  - Hypofractionation and high doses per fraction
  - *α*/*β* Model
  - Tumor control probability (TCP) versus normal tissue complication probability
  - Equivalent dose in 2‐Gy fractions, biologically effective dose
18. Radiation genetics: radiation effects on fertility and mutagenesis
  - Target cells for infertility
  - Doses to result in temporary and permanent sterility
  - “Reverse‐fractionation effect”
  - Mechanisms of mutation induction
  - Relative risk versus absolute risk
  - Time course and latency period/risks of cancer induction in different organs and tissues
  - Exposures, fertility, risks, and management strategies from preconception to birth
19. Molecular mechanisms
  - Molecular cloning techniques
  - Gene analyses
  - Oncogenes and tumor suppressor genes
20. Drug radiation interactions
  - Chemotherapy
  - Immunotherapy

#### 3.1.6 Anatomy and physiology

1. Language of anatomy
2. Planes
3. Projections
4. Imaging conventions
5. Landmarks
6. Homeostasis
7. Tissues, body membranes, and the skin
8. Epithelial tissue
9. Connective tissue
10. Tissue repair
11. Body membranes
12. Skin (cancer and radiation effects on the skin and hair)
13. Basic cytopathology
14. Musculoskeletal system
15. Anatomy and physiology of muscle tissue
16. Anatomy and physiology of bone tissue
17. Major bones and muscles (landmarks and features, imaging landmarks)
18. Joints
19. Imaging appearance—normal and common pathology
20. Endocrine system
21. Endocrine signaling (steroid hormones, peptide hormones, and second messengers)
22. Hypothalmic‐pituitary axis
23. Thyroid and its hormones
24. Parathyroids and their hormones
25. Thymus and immune system development
26. Pancreas and its hormones
27. Adrenal glands and their hormones
28. Ovaries and their hormones
29. Testes and their hormones
30. Imaging appearance—normal and common pathology
31. Nervous system
32. Nerves and microanatomy
33. Central nervous system (lobar and ventricular anatomy of the brain, functional areas of the brain (sensory and motor homunculus, thalamus, hippocampus, Wernicke's area, Broca's area, sensory cortices, chiasm, and pituitary), anatomy of the spinal cord and spinal nerves)
34. Peripheral nervous system (cranial nerves, dermatomes, and reflex arcs)
35. Human visual system (perception, integration time, contrast sensitivity, imaging implications for perception of medical images)
36. Functional classification
37. Imaging appearance: normal and common pathology on CT, MRI, and functional magnetic resonance imaging (fMRI)
38. Cardiovascular system
39. Blood and microanatomy (hematopoiesis and formed element lineages, vascular anatomy)
40. The heart (chambers, valves and their function, intrinsic conduction system, great vessels and their connection to the systemic circulation, coronary arteries, and coronary angiography)
41. Major vessels and circulations (aorta and its branches, cerebral circulation, portal vein and abdominopelvic drainage, major venous structures of abdomen/pelvis)
42. Physiology (vital signs, autoregulation)
43. Pulmonary system
44. Conducting zone (pharynx, larynx, trachea, and main bronchi)
45. Respiratory zone (lobar bronchi through respiratory bronchioles, blood–air interface, pulmonary ventilation, physics and physiology of gas exchange)
46. Thorax (external landmarks and links to bony and muscular anatomy, lung anatomy, normal, and common pathology on X‐ray and CT imaging)
47. Relationship with the cardiovascular system (pulmonary arteries and veins, physics and physiology of gas exchange in body tissues, blood pH, and the bicarbonate buffer system)
48. Lymphatic system
49. Lymph nodes, lymphatic vessels, and cisterna chyli
50. Lymphatic ducts and relationship with cardiovascular system
51. Regions of lymphatic drainage and relationship to cancer spread
52. GI system
53. Alimentary canal (oral cavity, teeth, salivary glands/parotid glands, salivary amylase, pharynx, esophagus, tunics of the alimentary canal, stomach, small intestine, and large intestine)
54. Peritoneum and mesenteric attachments
55. Accessory organs (liver, gallbladder, pancreas, and biliary system)
56. Digestion and its regulation (hormones, enzymes, and nervous system)
57. Imaging appearance: normal and common pathology
58. Urinary system
59. Kidneys (anatomy from nephron to renal tubule, glomerular filtration, tubular reabsorption, and secretion)
60. Physiology (electrolyte and blood volume regulation, renin‐angiotensin system, blood pH regulation, and the bicarbonate buffer system)
61. Collecting system (renal pelvis, ureters, urinary bladder, and urethra)
62. Imaging appearance: normal and common pathology
63. Reproductive system
64. Meiosis
65. Male (testes, duct system, prostate, external genitalia [penile bulb], and spermatogenesis)
66. Female (ovaries, uterus, external genitalia, ovarian cycle, uterine cycle, and mammary glands/breasts)
67. Conception and fetal development

#### 3.1.7 Mathematical and statistical methods

**Mathematical methods**

1. Math background
  - Fermi‐type estimation problems (e.g., how to estimate input data if it is not readily available and recognition of the uncertainties caused by these estimations)
  - The complex plane, odd/even functions
  - Algorithm complexity and basic linear algebra subroutines (BLAS)
  - Entropy and information gain
2. Introduction to linear systems
  - Fourier's theorem: Fourier series and the continuous Fourier transform
  - Properties of the Fourier transform
  - Gaussian, sinc, rect, sinusoid, and comb functions and essential Fourier transform pairs
  - Dirac delta function/the impulse symbol
  - Linear time‐invariant systems
  - Complex transfer function
  - Convolution principle
  - The edge response function
  - Auto and cross‐correlation
  - Linear independence and vector spaces
3. Discrete signal processing
  - Sampling theorem and Nyquist frequency
  - Discrete Fourier transform (DFT)
  - Fast Fourier transform
  - Apodizing and aliasing
  - Approximate restoration from sampling (pixels)
4. Mathematics of noise
  - Effect of noise on decision criteria
  - SNR
  - DQE and noise‐equivalent quanta
  - Principles of noise averaging, covariance concept
  - Autocovariance and power spectrum concepts
  - Filtering: inverse, Metz, Weiner, matched, and Wiener–Hellström filters
5. Mathematics of optimization
  - Cost functions
  - Unconstrained and constrained optimization
  - Convex optimization: simulated annealing and gradient approaches

**Statistical methods**

1. Descriptive statistics
  - Scales of measurement of observations: nominal, ordinal, interval, ratio
  - Univariate and multivariate observations
  - Distributions of observations (e.g., normal, binomial, lognormal, Poisson, and Gaussian)
  - Population parameters versus sample statistics
  - Distribution of statistics, random sampling
  - Graphical methods (e.g., box plots, probability plots, Loess plots, and time series)
  - Quality control statistics, univariate and multivariate control charts
  - Moments: expectation, mean, and variance
2. Probability
  - Classical
  - Bayesian
  - Random number generators, probability density, and distribution functions
3. Inferential statistics
  - Target population, sampled population, samples, and tolerance intervals
  - Distributions of sampling statistics: (e.g., chi‐squared, student's *t*, *F*)
  - Hypothesis testing, point and interval estimation, and resampling methods
  - Significance tests, level of significance as “associated probability”
  - Test of hypothesis (Neyman–Pearson) versus probability of hypothesis (Bayes)
  - Confidence intervals (Neyman–Pearson) versus credible intervals (Bayes)
  - Null and alternative hypotheses, multiple comparison problems (Neyman–Pearson), probability of hypothesis, likelihood ratios, Bayes’ factor (Bayes)
  - Type I and Type II errors, power of a statistical test
  - Sample size, power analysis
  - Propagation of error and the covariance matrix
  - Fourier relationships: characteristic function and the central limit theorem
4. Regression models
  - Linear regression models
  - Simple and multiple regression models
  - Logistic regression models
  - Log‐linear and Poisson models
  - Nonlinear models (nonlinear in parameters)
  - “Goodness‐of‐fit” measures (correlation coefficient)
  - Interpolation and extrapolation of models
  - Regularization: lasso and Tikhanov
5. Multivariate analysis
  - Cluster analysis
  - Discriminant analysis
  - Factor analysis
  - Principal component analysis
6. Categorical data analysis
  - Odds ratio and relative risk, attributable risk
  - Logit and log‐linear models
  - Receiver operating characteristic (ROC) analysis
  - Sensitivity, specificity, and predictive value
7. Design of clinical studies
  - Reliability and validity of a study, including internal and external validity
  - Random selection (population inference), random allocation (causal inference)
  - Design and analysis of randomized controlled studies, including strengths and weaknesses
  - Design and analysis of case‐control and cohort studies, including strengths and weaknesses
  - Data mining studies, including strengths (high external validity) and weaknesses (low internal validity)
  - Experimental design for multiple treatment groups consisting of different individuals
  - Experimental design for multiple treatments in the same individual

#### 3.1.8 Computational methods and medical informatics

**Computational methods**

1. Basic computer skills
  - Spreadsheet software, word processor, presentation software, and PDF editor
  - Search engine syntax, journals (e.g., Pubmed, Google Scholar)
  - Image viewing and processing software
2. Computer science
  - Data structure, memory, and ports
  - Bits, bytes, single and double precision
  - Debugging
  - High‐level language and editor (e.g., Python, Javascript, C/C++)
3. Programming Skills
  - Syntax
  - Data types (floating point variables, integers, strings, and arrays)
  - Declaration of variables
  - Logic, Boolean operators, switches, and conditional statements
  - Iterative programming
  - Methods, functions, programs, executables, compilation, and input/output
4. Machine learning
  - Supervised learning (classification and regression)
  - Unsupervised learning (principal component analysis and clustering)
  - Reinforcement learning
5. Particle transport
  - Linear Boltzman equation
  - Applications of Monte Carlo technique
  - Dose calculation algorithms: convolution and superposition

**Medical informatics**

1. Networking
  - Types of networks, data rate, and bandwidth
  - Network infrastructure
  - Wide area network, local area network
  - Communication protocols: TCP/IP and OSI Models
  - Cloud storage
2. Communication standards
  - Digital imaging and communications in medicine (DICOM)
  - Health Level Seven
  - Integrating the health‐care enterprise
3. DICOM and DICOM‐RT
  - Service object pairs, association negotiation
  - Information model elements (image, series, study, patient, instances, unique identifiers)
  - DICOM tags and modules
  - DICOM anonymization
4. Databases
  - Tables and fields
  - Database schema
  - Data dictionary
  - Queries and SEQUEL language
  - Client server database model
  - Backup
5. Picture Archive and Communication System (PACS)
  - Image storage
  - Image transfer—teleradiology
  - Image display—human visual system, ACR Technical Standard for Electronic Practice of Medical Imaging

#### 3.1.9 Research methods

1. Literature search and reading
2. Research methods and documentation
3. Academic writing, reviewing, and presentation
4. Clinical trials
5. Protocol and grant writing
6. Clinical translation and implementation
7. Laboratory management

#### 3.1.10 Professionalism (leadership, ethics, and communication)

**Leadership**

1. Personal and interpersonal
  - Emotional self‐awareness
  - Adaptability, initiative, and empathy
  - Organizational awareness and service orientation
  - Influence, conflict management, teamwork, and collaboration
2. Professional and developmental
  - Delegation and time management skills
  - Leadership communication skills
  - Professional vitality
  - New ventures leadership
  - Medical physics value and advocacy
3. Executive and administrative
  - Operations and finance
  - Information and human resources
  - Strategic planning
  - External affairs (external environment that affects the profession (e.g., regulatory and economic environment))

**Ethics and professionalism**

1. Ethical principles and values
2. Medical ethics
  - Ethical definitions and historical context
  - Ethics in the era of modern science
  - Nuremberg Code, Declaration of Helsinki, Belmont Report
3. Ethics in practice
  - Professional conduct
  - Clinical ethics
  - Research ethics
  - Education ethics
  - Business and government ethics
  - Public and professional responsibility, competence, and continuing education
  - Ethical encounters or dilemmas
4. Information privacy
  - Health Insurance Portability and Accountability Act (HIPAA)
  - Data security
5. Professionalism in practice
  - Roles and responsibilities of the medical physicist
  - Medical physics and related professional organizations, certification, and licensure
  - Interactions with other professionals
  - Responsible and ethical behavior
  - Diversity, equity, and inclusion

**Communication**

1. Scientific communication
  - Scientific writing
  - Scientific presentation
  - Public education
2. Clinical and professional communication
  - Written correspondence and reports, business proposals
  - Communication with other health‐care professionals
  - Communication with health‐care administrators
3. Patient‐centered communication
  - Establishing clinical relationships (physics‐patient consultation)
  - Verbal and nonverbal communication, active listening
  - Empathy, emotional status, and psychological considerations
  - Patient advocacy and communicating with families
  - Literacy, language, and cultural barriers

### 3.2 Additional topics (optional)

#### 3.2.1 Biology/oncology/medicine

#### 3.2.2 Advanced physics, engineering, computer science

#### 3.2.3 Frontiers in medical physics and opportunities outside of medical physics

1. Radiation Therapy
  - Radiomics, theranostics; connection to genomics and other ‐omics
  - Immunotherapy + RT
  - RT for non‐cancer treatments (e.g., cardiac ablation)
  - FLASH
  - Cerenkov imaging (applications for RT, proton and heavy ion therapy)
  - Chemotherapy + RT
  - PDT + RT
  - High‐intensity focused ultrasound + RT
  - Tumor‐treating fields
2. Diagnostic Imaging
  - Alternative X‐ray sources (synchrotron; inverse‐Compton scatter/laser‐particle accelerators)
  - Radiomics, theranostics; connection to genomics and other ‐omics
  - CAD and AI/machine learning (beyond the basics)
  - X‐ray interferometry
  - Nanomedicine (contrast agents)
  - Photon counting X‐ray detectors
  - Biophotonics
  - Magnetoencephalography
3. Nuclear Med
  - Theranostics
  - Nanomedicine (radiotracers for imaging and/or therapy)
  - Radiotracer development/radiochemistry
4. Medical health physics
  - Personalized dose calculations
5. Opportunities in other medical specialties
  - Surgery: image‐guided surgery, surgical planning based on imaging, augmented reality, and virtual tools
  - Pathology: automated analysis, pathology image display and archiving
  - Ophthalmology: laser surgery, optical modeling, and corrective optics
  - Dentistry: mechanical modeling, 3D planning, and 3D optical reconstruction
  - Orthopedics: mechanical modeling, motion analysis, and hardware design
  - Cardiology: electrophysiology, mechanical modeling, and flow modeling
  - Neuroscience, psychology, and psychiatry (e.g., incorporation of imaging in diagnosis and monitoring)

#### 3.2.4 Business aspects of medical physics

1. Clinical
  - Medical physics–specific charges
  - Clinical roles and operation (from a business perspective)
  - Systems‐based practice
  - Licensing (differences between states)
  - Purchase agreements (e.g., service and support contracts)
2. Financial principles
  - Billing and revenue (professional and technical codes)
  - Equipment purchasing (selection, revenue generation, and budget management)
  - Fiscal statements
  - Financial/market strategy
3. Accounting principles
  - Accounting standards
  - Business models, pro‐forma
4. Business aspects of grant management
5. Regulatory compliance and policy in medical physics
6. Legal principles (contracts and agreements, tort law, liability, and introduction to civil procedure)

#### 3.2.5 Teaching for medical physicists

1. Best teaching practices
  - Traditional pedagogies
  - Assessment and evaluation
  - Teaching critical thinking
  - Clinical teaching and mentoring
  - Online teaching and learning
  - Blended learning
  - Open textbooks and online resources
2. Cognitive science
  - Adult learning theory
  - Bloom's taxonomy
  - Metacognition
3. Active Learning
  - Peer instruction
  - Just in time teaching
  - The flipped classroom
  - Project‐based learning

### 3.3 Diagnostic imaging specialization (optional)

1. Radiation physics and detection for imaging
  - Core topics from radiological physics and dosimetry (Section 3.1.1)
    - Photon interactions relevant to imaging
    - Radiation detectors used in imaging
2. Foundations of imaging science
  - Deterministic and stochastic description of imaging systems, objects, and images
    - Core topic of linear systems in mathematical and statistical methods (Section 3.1.7.)
    - Linear and nonlinear systems: discrete versus continuous
    - Stochastic models of objects and images
    - Transport theory and diffraction theory for imaging
    - Mathematical description of noise in imaging systems (Poisson statistics, shot noise, and speckle)
  - Core topics in computational methods and medical informatics (Section 3.1.8)
  - Digital representation of images
  - Human visual system and perception
  - Display of images
    - Display hardware
    - Grayscale rendition, ambient illumination, maximum, and minimum luminance
    - Spatial rendition
    - Color rendition
3. Digital image processing
  - Core topics from mathematical and statistical methods (Section 3.1.7)
    - Discrete signal processing
  - Pixel‐based operations
    - Windowing and leveling; thresholding and binarization; and image subtraction
    - Histogram equalization
  - Convolution and spatial domain filtering
    - Smoothing/averaging, median filtering, unsharp masking
  - Fourier‐space filtering
    - Low‐pass, band‐pass, and high‐pass
  - Coordinate and affine transformations
    - Magnification and interpolation
    - Image deformation
    - Image registration: mutual information, mean square distance, normalized cross‐correlation
    - Deformable image registration: B‐spline, intensity‐based
    - Image agreement metrics: Dice similarity coefficient and Hausdorff distance
    - Image fusion and display
  - Segmentation
  - Analysis
    - Similarity, cross‐correction
    - Texture, shape
    - Neural networks and machine learning; computer‐aided diagnosis
  - Compression, lossy and lossless methods
  - Imaging informatics
    - Core topic of medical imaging informatics from computational methods and medical informatics (Section 3.1.8)
    - Standardization of digital file formats; DICOM and other standards encountered in imaging
    - PACS, radiology information systems, and electronic medical records; related health‐care information systems
    - Protection of information, HIPAA
    - Anonymization for research
4. Image quality
  - Resolution/unsharpness/blur, PSF, line‐spread function (LSF), edge‐spread function (ESF), MTF
    - Spatial domain metrics: PSF, LSF, and ESF
    - Fourier domain metrics: MTF
  - Contrast
  - Noise (statistical, structured); NPS
  - Composite metrics
    - SNR, CNR, and contrast detail
    - DQE
  - Perceptual metrics
    - Detectability
    - Task‐based observer performance (false/true positive/negative; sensitivity, specificity, accuracy; positive predictive value, precision and recall, *f*‐score)
    - Ideal and other numerical observers
    - ROC analysis, applications, and extensions
5. Image reconstruction
  - Inverse problems
  - Projection geometry in 2D
    - The Radon transform and the sinogram
    - Parallel‐beam and fan‐beam geometry
  - Relationship of Radon and Fourier transforms: the central slice theorem
    - Image reconstruction based on interpolation and inverse Fourier transform
  - Analytical reconstruction
    - Backprojection
    - Ramp filter and filtered backprojection
    - Noise‐reduction filters
    - Inverse Radon transform
  - Extension of projection geometry and reconstruction to 3D and 4D
    - Central plane theorem and fully 3D reconstruction algorithms
    - Cone‐beam geometry and reconstruction algorithm
    - Gating and binning of 4D data
  - Iterative reconstruction
    - Advantages and limitations compared to analytical methods
    - Forward (physics) model
    - Cost functions
    - Classes of iterative algorithms
    - Common algorithms and applications
  - Image registration in sinogram space
6. X‐ray production
  - History: Roentgen's discovery of X‐rays; evolution from cathode‐ray tube (Coolidge)
  - Core topics from radiological physics and dosimetry (Section 3.1.1)
    - Characteristic radiation
    - Bremsstrahlung production
  - Generic hot‐cathode X‐ray tube design (Coolidge design)
    - Filament, target, and focal spot
    - Anode materials; beveled and rotating anodes
    - Ancillary components for filtration and collimation, cooling, shielding
  - Cold‐cathode X‐ray tube design
  - High‐voltage generators
    - Transformers and rectification
    - Three‐phase, multiphase, and ripple
  - Impact of design and operating parameters
    - Filament current and tube current
    - Accelerating voltage
    - Exposure time
    - Characteristic and bremsstrahlung emission spectra
    - Heating and cooling
    - Heel effect
  - Requirements for specific applications (mammography, CT, and fluoroscopy)
7. X‐ray detectors
  - Radiographic film and radiochromic film
    - Composition of emulsion
    - Development
    - Characteristic (H&D) curve, speed, and latitude
  - Intensifying screens
    - Composition
    - Influence on blur, efficiency, and patient dose
  - Storage phosphor plates
    - Composition
    - Computed radiography readout process
  - X‐ray flat‐panel detectors
    - Indirect versus direct conversion
    - CCD and CMOS technologies
    - Scintillators
  - Gas‐filled detectors for CT
8. Projection X‐ray Imaging
  - Radiography
    - Projection geometry
    - Techniques (acquisition parameters and protocols)
    - Magnification; source‐to‐image distance, source‐to‐object distance
    - Anti‐scatter grids and other scatter‐reduction techniques (air gap, slot scan)
    - Collimators, automatic exposure control, and ancillary devices
    - Impact of techniques on image quality, artifacts; appropriate technique choices
    - Impact of techniques on dose (dose vs. kV, mA s, magnification); appropriate technique choices
    - Contrast agents, temporal subtraction imaging
    - Dual‐energy imaging, tissue/energy‐selective imaging, photon counting
    - Portable radiography
  - Mammography
    - MQSA and related regulatory requirements
    - Mammography‐specific concerns: resolution and contrast versus dose; screening mammography versus diagnostic mammography
    - Mammography‐specific choices: X‐ray source and filtration; geometry and collimation; generator
    - Mammography‐specific detector requirements
    - Techniques: compression, scatter reduction, magnification; automatic exposure control
    - Image quality, artifacts and dose
    - Digital mammography
    - Breast tomosynthesis
    - Computer‐aided diagnosis in mammography
  - Fluoroscopy
    - Fluoroscopy‐specific concerns: resolution and contrast versus dose
    - Fluoroscopy source requirements (vs. radiography)
    - Image intensifier (II) design and operation; II‐specific image quality issues
    - Flat panel detectors as alternative to an image intensifier
    - Modes of operation; control curves; added filtration
    - Contrast agents and subtraction imaging techniques: temporal and, dual energy
    - Image quality, artifacts, and dose, including to personnel
    - Interventional radiology: angiography, cardiac catheter lab, and intraoperative
9. Volumetric X‐ray imaging
  - Computed tomography
    - History: Hounsfield, Cormack
    - CT geometries (rotating vs. stationary components; fan‐beam vs. parallel; multidetector and cone beam)
    - Filtration/compensation, collimation, and scatter reduction
    - CT detectors (gas and solid‐state designs)
    - Techniques (kV, mA, rotation speed, slice thickness, pitch) and acquisition modes (sequential, helical, and scout/scanned projections)
    - Contrast agents
    - Impact of techniques on image quality, artifacts; appropriate technique choices
    - Impact of techniques on dose; CT dosimetry (computed tomography dose index [CTDI], dose length product [DLP], effective dose); appropriate technique choices, dose versus risk
    - Specific applications (cardiac, lung, including gating/4D; dual‐energy; perfusion)
    - Utilization with functional modalities (PET, SPECT)
  - Cone‐beam CT
    - Acquisition geometry (differences vs. CT)
    - Image reconstruction (differences vs. CT)
    - Image quality, artifacts, noise, and dose
    - Applications (onboard imaging for RT; dedicated imaging for cardiac, intraoperative, dental, musculoskeletal/extremities)
  - Tomosynthesis
    - Acquisition geometry; evolution from classical tomography
    - Image reconstruction
    - Image quality versus CT and radiography
    - Patient dose versus CT and radiography
    - Applications to breast, lung, and other organs
  - Dual‐energy X‐ray absorptiometry
    - Bone mineral density image derivation
    - Projection geometry
    - Techniques (acquisition parameters and protocols)
    - Other applications (body composition, vertebral fracture, and abdominal aortic calcification)
10. Ultrasound imaging
  - Ultrasound physics (speed, impedance; reflection/refraction/scattering/attenuation) and propagation (intensity/pressure vs. distance/laterally; near‐field, far‐field)
  - Transducers (physics, materials; design and operation)
  - US systems (ancillary components) and operation (focusing and steering; gain and attenuation compensation)
  - Contrast agents
  - Echo 2D imaging
    - Acquisition methods
    - Image display
    - Real‐time imaging and image quality
  - Image quality: resolution (axial, lateral, elevational), artifacts, noise
  - Harmonic imaging
    - Acquisition methods
    - Image display and image quality
  - Elastography
    - Acquisition: static compression, transient elastography, and acoustic radiation force impulse methods
    - Analysis: characteristics of tissue stiffness and correlation with disease processes
  - 3D imaging
    - Acquisition methods
    - Image display and image quality
  - Doppler flow measurement; Doppler imaging
    - Hardware and operation; data analysis
    - Operating modes (continuous, pulsed; duplex; color flow)
    - Data quality and artifacts
  - Transmission ultrasound imaging
    - Acquisition methods; image processing
    - Image quality
  - Bioeffects and safety
    - Bioeffects; relationship to US power
  - Non‐imaging (therapeutic) applications of US
    - Ablation, lithotripsy, and heat (warming) therapy
11. Magnetic resonance imaging
  - History (e.g., Bloch, Purcell, Lauterbur)
  - Underlying physics (magnetism, magnetic moments of nuclei, and induction)
  - Physics of nuclear magnetic resonance (precession and resonance, energy absorption/emission, excitation, relaxation, and dephasing)
    - Precession and resonance; Larmor frequency; effect of *B*‐field strength
    - Energy absorption/emission
    - Excitation
    - Relaxation (T1, T2) and dephasing (T2*)
  - Nuclear magnetic resonance (NMR) pulse sequences
    - Hardware (coils and magnets)
    - Excitation pulses and gradients
    - Timing diagrams
    - Rephasing and echoes
    - Weighting
    - Specific pulse sequences (spin echo, inversion, and gradient echo)
  - Contrast agents
  - MR spatial signal localization
    - Gradient coils (hardware; types: slice, frequency, phase; rephasing)
    - Timing diagrams
    - *k*‐Space: relation to spatial domain via Fourier transform
    - *k*‐Space filling strategies
  - MR image acquisition modes (for each: timing diagram, acquisition time vs. time to echo, time to repetition, other parameters)
    - 2D spin echo
    - Inversion recovery
    - 2D multiplanar
    - Fast spin‐echo
    - Gradient echo
    - Echo planar
    - Parallel imaging
    - 3D FT
  - Image quality (including effects of acquisition parameters)
  - Artifacts (e.g., instrumentation, motion, susceptibility, and molecular environment)
  - Special techniques
    - Spectroscopy
    - Angiography
    - Elastography (liver, brain)
    - Perfusion and fMRI
    - Diffusion weighted imaging
    - Chemical exchange saturation transfer and magnetization transfer
    - Quantum coherence
  - MR bioeffects
  - Principles of MR safety (including aspects of personnel and patient safety)
  - Siting and facility/QA and regulatory requirements
12. Quality management in DI
  - Regulations and recommendations
  - Process mapping
  - Failure modes and effects analysis
  - Fault tree analysis
  - Establishing a quality management program
13. Radiation protection in imaging
  - Core topics from radiological physics and dosimetry (Section 3.1.1) and radiation protection and safety (Section 3.1.2)
  - Entrance air kerma, entrance exposure; relationship to dose and dose equivalent
  - Radiation dose and risk
    - Typical whole‐body dose values for imaging procedures
    - Limiting tissues/organs for imaging of different anatomical regions
    - Potential for dose to uterus and fetus from imaging procedures
    - Stochastic and deterministic effects expected from imaging procedures
  - Optimization of dose in imaging
    - “Right‐sizing” techniques
    - Image gently, image wisely
  - Radiation protection of personnel
    - Personnel‐protective equipment, including dosimeters
    - Utilization of time, distance and shielding for protection
    - Shielding design: NCRP 147 and TG 108
14. Practical/laboratory training in DI
  - For each modality:
    - Scanner operation (including imaging of phantoms and image analysis to learn about acquisition parameters vs. image quality/artifacts)
    - QA (procedures, QA phantoms, data processing/analysis)
    - Radiation protection (shielding calculations and assessment; personnel and area monitoring)
    - Safety (interlocks, signage)
  - Dose measurement:
    - Projection X‐ray imaging: measurement of entrance air kerma; influence of acquisition parameters; dose area product (kerma area product) and peak skin dose considerations; relevant TGs
    - CT: measurement of CTDI, DLP; influence of acquisition parameters; size‐specific dose estimate; relevant TGs
  - Observation/shadowing
    - Reading room observation and interaction
    - Technologist observation and interaction

### 3.4 Nuclear medicine specialization (optional)

1. Basic physics
  - Basic atomic and nuclear physics
  - Modes of radioactive decay
  - Radioactive decay rates, including transient and secular equilibrium
  - Passage of radiation through matter
2. Radiation detection
  - Scintillation
  - Photomultiplier tube and solid‐state light detection
  - Ionization‐based detection—gas and solid state
  - Instrumentation for radiation detection
  - Signal propagation
  - Counting statistics
  - Propagation of error
3. Clinical applications of NM
  - Radiotracer methods and the breadth of applications
  - Required scanning modes: dynamic, static, whole‐body, gated
4. Radionuclide production
  - Cyclotron and targetry principles
  - Reactor‐based production
  - Generators (Mo99/Tc99, Ge68/Ga68, Sr82/Rb82)
  - Radionuclide QC
5. Radiotracer production
  - Principles of radiochemistry
  - Radiopharmacy
  - Radiopharmaceutical QC
6. Radioactivity measurement devices—principles and applications
  - Dose calibrator (activimeter)
  - Well counter
  - Thyroid uptake probe
  - Liquid scintillation counter
7. Conventional gamma camera
  - Design and components
  - Energy and position measurement
  - Collimators
    - Principles
    - Sensitivity versus resolution
    - Photon energy—septal penetration
    - Geometry—parallel hole, fan‐beam, cone‐beam, and pinhole
  - Corrections—linearity, energy, and uniformity
  - Attenuation and scatter effects
  - Measures of intrinsic and extrinsic performance
8. Image reconstruction
  - The detection model
  - Analytic methods, including filtered backprojection
  - Iterative methods, including maximum likelihood expectation maximization and ordered subsets expectation maximization
  - Noise characteristics
  - Sampling considerations
  - Incorporating physical effects (e.g., attenuation, spatial resolution) into the reconstruction model
  - Other means of correcting degrading effects
  - Methods of noise control—low‐pass filtering, limiting iterations, and regularization
  - Evaluating reconstructed image quality
9. SPECT
  - Acquiring SPECT projections with a gamma camera
  - Performance requirements—center of rotation and uniformity
  - SPECT attenuation and scatter correction methods
  - SPECT/CT
  - CT‐based attenuation correction, SPECT, and CT fields‐of‐view registration
10. PET
  - Positron imaging physics
  - Coincidence detection
  - Basic detector geometry
  - Sensitivity and corrections (normalization, timing, energy, and position maps)
  - Scattered and random events, random estimates
  - Noise‐equivalent counts and statistical quality of data
  - Time‐of‐flight PET—principles, benefits
  - Attenuation—qualitative and quantitative effects
  - Attenuation correction
  - Detector design
    - Scintillators
    - Silicon photomultiplier detectors
    - Blocks versus one‐to‐one coupling
    - Dead time
    - Resolution, energy, and timing resolution
  - Factors limiting intrinsic resolution
  - PET/CT
    - CT‐based attenuation correction
    - Model‐based scatter correction
    - PET and CT fields‐of‐view registration
11. Other imaging systems
  - PET/MR
  - Whole‐body PET/CT systems
  - Novel SPECT designs
  - Dedicated cardiac SPECT systems
  - Dedicated breast systems (PET and single‐photon)
  - Unique challenges with corrections and QC
12. Computer principles in NM
  - Data storage formats
  - Digital image representation
  - Basic image manipulation and processing
  - Image display—intensity settings and color scales
13. Quantitative imaging techniques
  - Concepts
  - Planar image quantitation—methods and fundamental limitations
  - Radionuclide quantitation in SPECT and PET
  - Requirements for accurate quantitation
  - Standardized uptake value and related parameters
  - Region‐of‐interest analysis techniques
  - Time–activity curve analysis techniques
14. Tracer kinetics
  - Basic concepts of radiotracers and tracer kinetics
  - Tracer kinetic modeling
15. Radiation dosimetry for NM
  - Patient considerations
  - Staff considerations
  - General public considerations
  - MIRD method
  - Image‐based dosimetry
16. Therapeutic NM
  - Therapeutic radionuclides
  - Alpha versus beta versus gamma dosimetry
  - Currently used therapeutics
  - Radiation protection in radionuclide therapy
  - Patient‐specific dose determinations/dosimetry‐based treatment planning
  - Theranostic—imaging/therapy pairs
  - Imaging therapeutic radionuclides
17. Radiation protection for NM
  - Core topics from radiation protection and radiation safety (Section 3.1.2)
  - NM shielding
    - Multiple sources
    - Fraction emitted from patient
    - Decay factor during uptake and imaging
    - Broad beam fitting parameters and transmission
  - CT component for shielding
  - NM‐specific radiation protection and safety, including room and personnel surveys, shielding design/evaluation, protection devices (syringe shields, L‐blocks)
  - Patient as source, hazards to staff, release criteria/regulations and calculations
18. Quality management in NM
  - Dose Calibrator QC tests: accuracy, linearity, geometry, constancy, and special cases (e.g., pure beta emitters, therapy radionuclides for which there is not a factory setting, PET radionuclides for quantitative accuracy)
  - Well counter/thyroid uptake counter QC tests: chi‐squared, constancy, efficiency, and uptake accuracy
  - Planar gamma camera QC: extrinsic and intrinsic uniformity, resolution and linearity, energy resolution, count rate performance, multiple‐window spatial registration, sensitivity, and NaI(Tl) crystal hydration
  - SPECT QC: high contrast resolution, cold sphere contrast, uniformity, and center of rotation
  - PET QC: well counter calibration, sensitivity, count rate performance, resolution, quantitative accuracy, hot and cold sphere (or cylinder) high contrast detectability, and uniformity
  - CT QC: image registration, Hounsfield unit accuracy, uniformity, low‐ and high‐contrast resolution, and dosimetry
  - RAMs program review/audit
    - Department of Transportation (DOT) training and placarding
    - Incoming and outgoing packages
    - Area radiation surveys
    - Area wipe‐testing
    - Waste storage and disposal
    - Patient dosing records
    - Radionuclide therapy record keeping
  - Process mapping
  - Failure modes and effects analysis
  - Fault tree analysis
  - Establishing a quality management program
19. Practical/laboratory training in NM
  - Reading room observation and interaction
  - Technologist observation and interaction
  - Radionuclide handling and hot lab skills
  - Radioactive spill management
  - Phantom preparation
  - Scanner operation—scanning and image processing
  - Radionuclide generator observation
  - NM therapy observation

### 3.5 Radiation therapy specialization (optional)

1. Clinical radiation oncology
  - Cancer etiology, classification, and staging
  - Cancer statistics (incidence, survival and hazard functions, proportional hazards model, and Kaplan–Meier analysis)
  - Overview of oncology treatment modalities
  - Core topics from radiobiology (Section 3.1.5) and anatomy and physiology (Section 3.1.6)
  - Radiobiological basis of RT
  - Workflow of clinical oncology and radiation oncology
2. Physics and operation of radiation oncology equipment
  - Linear accelerators (theory, components, commissioning, and QA, safety)
  - Other external beam RT equipment (isotope units, cyclic accelerators, and accelerators for particle beam therapy)
  - Brachytherapy equipment (afterloaders, applicators, electronic brachytherapy units, commissioning, QA, and safety)
  - Imaging equipment in RT (CT, MRI, PET, and onboard imaging)
  - Computerized treatment planning systems (commissioning, QA, and maintenance)
3. Ancillary physics equipment (patient immobilization, dosimetry, QA equipment)
  - External beam RT
  - External beam RT modalities (properties/characteristics of photon, electron, and heavy charged particle beams)
  - Beam output calibration
  - Target definition, treatment intent, and dose prescription criteria
    - Treatment simulation techniques
    - Patient data acquisition: non‐imaging to imaging‐based
    - Immobilization devices and techniques
    - Motion management techniques
  - Prescribing, reporting, and evaluating RT treatment plans
    - Prescription and reporting nomenclature
    - Margins, uncertainties, and accounting for patient motion
    - Treatment plan evaluation and metrics
  - Treatment planning techniques
    - Dose specification and normalization
    - Isodose distribution for static fields in reference conditions
    - Corrections for dose calculations in a patient
    - Beam orientation and modification (collimation, multi‐leaf collimator, wedges, applicators, and bolus)
    - Manual and computerized dose calculation algorithms
    - Forward and inverse planning/treatment plan optimization, and plan robustness
    - Radiobiological considerations in treatment planning and plan optimization
  - Photon‐beam treatment planning
    - Energy and field size selection
    - Modulated delivery techniques
  - Electron‐beam treatment planning
    - Energy and field size selection
    - External shielding, internal shielding, and backscatter dosimetry
  - Particle‐beam treatment planning
    - Particle type, energy, and field size selection
    - Delivery and optimization techniques
    - Field matching and patch fields, range uncertainties, and plan robustness
    - Estimation of RBE and positional variations
  - QA and safety
    - Initial chart check and continuous review
    - Patient‐specific QA
  - Treatment delivery and verification
    - Setup and immobilization
    - Image guidance
    - Adaptive radiation therapy (ART)
    - In vivo dosimetry
4. Brachytherapy
  - Radioactive sources
    - Types of sources
    - Source strength specification
    - Calibration equipment
  - Treatment planning and dose specification
    - Radiobiological considerations in brachytherapy
    - Implant technique
    - Source loading techniques
    - Dose calculation techniques
    - Prescribing, reporting, and evaluating brachytherapy treatments
    - Disease site–specific planning techniques
    - Imaging guidance systems
  - Treatment delivery techniques
    - Permanent implant brachytherapy
    - Afterloader‐based brachytherapy
    - Unsealed radionuclide therapy
    - Intraoperative brachytherapy
    - Electronic brachytherapy
  - QA and safety
    - Routine QA
    - Brachytherapy room and hot lab shielding
    - Receiving and shipping of RAMs
    - Regulatory compliance for RAMs
5. Special techniques in RT
6. Rationale for development of special techniques, required physical, and staffing resources
7. TBI
8. Total skin electron irradiation
9. Intraoperative RT
10. SRS
11. Stereotactic body radiation therapy, stereotactic ablative radiotherapy
12. Hyper‐ and hypofractionation
13. ART
14. Electron arc therapy
15. Hyperthermia
16. Imaging for RT
17. Core topics from fundamentals of imaging in medicine (Section 3.1.3)
18. Treatment simulation processes (including CT simulation, MR simulation, 4DCT)
19. Multimodality imaging for treatment planning
20. Image registration and deformable image registration
21. Imaging for treatment guidance and verification
22. Imaging for motion management (including surface imaging techniques)
23. Imaging for ART
24. Imaging for treatment response
25. Radiation protection in RT
26. Core topics from radiation protection and radiation safety (Section 3.1.2)
27. Operational safety guidelines
28. Radiation protection programs
29. Structural shielding
30. Quality management in RT
31. Regulations and recommendations
32. Process mapping
33. Failure modes and effects analysis
34. Fault tree analysis
35. Establishing a quality management program
36. Practical/laboratory training in RT
37. Overview of clinical radiation oncology QA/QI/peer review
38. Measurement of absorbed dose
39. QA (procedures, machine‐specific, and patient‐specific)
40. Treatment planning (hardware, software, objectives, and techniques)
41. Brachytherapy (planning, delivery, and QA)
42. Radiation protection (shielding, personnel monitoring, and measurement techniques)

### 3.6 Medical health physics specialization (optional)

1. Biological effects of radiation
  - Core topics from radiobiology (Section 3.1.5) and radiation protection and radiation safety (Section 3.1.2)
  - Patient dose assessment and dose reconstruction (examples): for example, fluoroscopic skin dose, fetal dose, and RAM administrations
  - Patient dose tracking (examples): regulatory and accreditation requirements, significant radiation dose level, and diagnostic reference levels
2. Advanced radiation detection
  - Core topics from radiological physics and dosimetry (Section 3.1.1) and radiation protection and radiation safety (Section 3.1.2)
  - Laboratory instrumentation: well counters, high purity germanium, liquid scintillation counting
3. Advanced internal dosimetry
  - Inhalation, ingestion, and injection models
  - Modeling organ clearance, effective half‐time, and curve fitting
  - MIRD formalism, applications in radionuclide therapy
  - Phantoms: mathematical models and physical phantoms
  - Staff monitoring: committed effective dose equivalent, bioassays, air limits, derived air concentrations
4. Advanced external dosimetry
  - Point, line, and volume gamma sources
  - Mathematical modeling of external dose distributions
5. Advanced radiation protection
  - Core topics from radiation protection and radiation safety (Section 3.1.2)
  - Principles—controlled versus uncontrolled areas, occupancy factors, and distances
  - Shielding evaluation techniques
  - Radiation safety with unsealed therapeutic RAM use
6. Regulatory oversight
  - Limited/broad scope materials licensing
  - X‐ray regulatory oversight (10 CFR (Code of Federal Regulations (United States)), US Food and Drug Administration [FDA], and state authority)
  - Patient release criteria and calculation techniques
  - Security requirements for sources
  - Radiation safety officer, authorized user, authorized medical physicist, and authorized nuclear pharmacist requirements
  - Medical event investigation/reporting
  - Root cause analysis
  - Six sigma training
  - Voluntary regulatory oversight (e.g., ACR, TJC)
  - Non‐RAM hazardous materials
7. Environmental monitoring and waste release
  - Release of radionuclides to the environment (air and sewer), air sampling
  - Dosimetric consequences of environmental release
  - Environmental Protection Agency (EPA) and NRC air and water dispersion models
  - High level, transuranic, and low level waste disposal, nuclear fuel cycle
  - USNRC/USDOE/USEPA repository (NRC/Department of Energy/EPA)
  - Low‐level compacts
  - Future impacts
8. Nonionizing radiation
  - Core topics from fundamentals of imaging in medicine (Section 3.1.3)
  - UV devices
  - Lasers: applications, laser safety officer, ANSI guidance
  - Radiofrequency and microwave radiation
9. Emergency Response
  - History of major accidents
  - Integration into broader emergency response community
  - Communication and mitigation
  - Reporting requirements
10. Nonclinical medical health physics
  - Radionuclides in research (human and animal)
  - X‐ray units in research (human and animal)
  - Industrial applications: irradiators, particle accelerators, and others
11. Quality management in medical health physics
  - Core topics from radiation protection and safety (Section 3.1.2)
  - Lead apron integrity assessment and tracking
  - RAMs program review (incoming and outgoing material, patient dosing records, and waste)
  - Unsealed RAM therapies
  - Communication of risk to patients and staff
  - Radiation safety in‐service training
  - Process mapping
  - Failure modes and effects analysis
  - Fault tree analysis
  - Establishing a quality management program
12. Practical/laboratory training in medical health physics
  - Safe radionuclide handling techniques, hot lab safety, personnel decontamination, and spill decontamination
  - Counting device characteristics: background rate, minimum detectable activity, energy resolution, efficiency, Chi‐squared, and dead time
  - Multichannel analyzer/gamma spectroscopy: photopeak, Compton edge, escape peaks, and backscatter
  - Gas chamber detector experiments, voltage curves
  - Area surveys, wipes, and sealed source/contamination wipe testing
  - Well counter QA testing
  - Dose calibrator QA testing
  - Lead apron evaluation
  - Evaluation of room shielding
  - Contamination surveys
  - Decommissioning and other decontamination techniques

### 3.7 Industry specialization (optional)

**Product creation**

1. Market research and strategy
  - Strategic marketing (e.g., vision, mission, purpose, strategy, objective)
  - Global RT and radiology demands
  - Industry trends/direction
2. Product creation process
  - Product creation process types
  - Phases of product creation process types
  - Key influences on production creation process
  - Project management tools (e.g., project management methodologies, and software)
3. Risk assessment
  - Risk assessment methodology in product design, manufacture, and deployment
  - Industry risk assessment focus areas
  - Regulatory implications on risk assessment
4. Design and manufacture
  - Hardware manufacturing methodology
  - Software development methodology
  - QA methods and systems
  - Manufacturing processes and systems

**Regulatory and legal**

1. Regulatory
  - Federal and state regulations on radiation‐producing devices and nuclear byproducts
  - Regulatory requirements and public safety
  - Regulatory requirements on X‐ray imaging and mammography systems
  - Storage, handling, and transport of nuclear byproducts
  - FDA clearance, approval, and medical device classes
  - 510(k) premarket notification and substantial equivalence
  - Compliance controls
2. Compliance
  - Compliance, especially regarding patient and staff safety
  - AdvaMed and US Manufacturer Code of Ethics
  - Role of National Institute of Standards and Technology as a regulator of industry standards to medical physics
  - Role of regulating bodies (e.g., NRC, FDA, and IAEA)
  - Role of accreditation commissions (TJC, CAMPEP)
  - Role of professional societies (AAPM, American Society for Radiation Oncology, Radiological Society of North America [RSNA], and Society of NM and Molecular Imaging)
  - NEMA and MITA standards
  - Common medical regulations (HIPAA, 10 CFR, 21 CFR)
  - Purpose and ubiquity of the DICOM standard
  - Data privacy and data protection
  - State‐specific data protection rules
  - Clinical QA and quality control
3. Legal considerations
  - Medical care corruption and anti‐corruption legislation
  - Foreign Corrupt Practices Act
  - Anti‐Kickback Statute
  - Sunshine Act
  - Whistleblower protections and ombudsmen
  - 501(c)(3) institution benefits and legal significance

**Policy in medical physics**

1. Policy and government
  - Definitions of policy, politics, policy analysis, advocacy, and activism
  - Types of policy (public, social, health, institutional, and organizational)
  - Spheres of political action in medical physics (government, workplace, professional societies, and community)
  - Structure, processes, and power of all three branches of the US government
  - Enactment of laws (bills and resolutions, authorizing vs. appropriating legislation)
  - Litigation and liability in the context of medical physics
  - Role of state governments in certification of medical physicists
  - International health‐care strategy (e.g., role of IAEA, World Health Organization)
2. Policy for science
  - Allocation of funding by Congress to relevant government entities (e.g., NIH, NSF, DOD, DOE)
  - Allocation of funding by government departments to specific research projects
3. Finance and Insurance
  - Structures of health‐care systems, their expenditures, and health outcomes (Extrapreneurial (US), Mandated Insurance (AUS, CAN), National Health Service (UK))
  - Sources of funding for the US health‐care system (e.g., Federal: Medicare, VA; State: Medicaid; Private: Employer‐based insurances)
  - Health‐care procedure coding, coverage, and payment
4. Further applications in medical physics
  - Current regulatory affairs landscape surrounding medical physics in the United States (e.g., professional licensure, use of medical devices and equipment)
  - Influence of professional societies on policy changes (e.g., AAPM committees, ACR, and federal/state advocacy)
  - Certification (ABR, American Board of Medical Physics, American College of Medical Physics, Canadian College of Physicists in Medicine, and quality management program or qualified medical physicist) policy and its effect on the field of medical physics
  - Other health policies pertaining to medical physicists, radiation oncology, or radiology (e.g., weighing in on national guidelines)

*Note: Some titles listed here are out of print but have been retained in this list due to their important historical role in medical physics education. Every effort has been made to list additional alternatives to these texts and instructors are encouraged to assure that students have access to all texts recommended by the instructor for a given course*.

## 4 BIBLIOGRAPHY

### 4.1 Core topics

#### 4.1.1 Radiological physics and dosimetry

- Evans RD. *The Atomic Nucleus*. McGraw‐Hill Company; 1955.
- Johns HE, Cunningham JR. *The Physics of Radiology*. 4th ed. Charles C Thomas; 1983.
- Knoll GF. *Radiation Detection and Measurement*. 4th ed. John Wiley & Sons; 2010.
- Podgorsak EB. *Radiation Physics for Medical Physicists*. Springer‐Verlag; 2005.
- Attix FH. *Introduction to Radiological Physics and Radiation Dosimetry*. John Wiley & Sons; 2008.
- Andreo P, Burns DT, Nahum AE, Seuntjens J, Attix FH. *Fundamentals of Ionizing Radiation Dosimetry*. John Wiley & Sons; 2007.
- Turner JE. *Atoms, Radiation, and Radiation Protection*. 3rd ed. Wiley; 2017.

#### 4.1.2 Radiation protection and radiation safety

- Hall EJ, Giaccia AJ. *Radiobiology for the Radiologist*. 8th ed. Lippincott Williams & Wilkins; 2018.
- National Research Council. *Health Risks from Exposure to Low Levels of Ionizing Radiation: BEIR VII Phase 2*. The National Academies Press; 2006.
- National Research Council. *Health Effects of Exposure to Radon: BEIR VI*. The National Academies Press; 1996.
- Cember H. *Introduction to Health Physics*. 3rd ed. McGraw‐Hill; 1996.
- Attix FH. *Introduction to Radiological Physics and Radiation Dosimetry*. John Wiley & Sons; 2008.
- Knoll GF. *Radiation Detection and Measurement*. John Wiley & Sons; 2010.
- Stabin MG. *Radiation Protection and Dosimetry: An Introduction to Health Physics*. Springer Science & Business Media; 2007.
- McGinley PH. *Shielding Techniques for Radiation Oncology Facilities*. Medical Physics Publishing; 1998.
- American Society for Radiation Oncology. *Safety Is No Accident – A Framework for Quality Radiation Oncology Care*. American Society for Radiation Oncology; 2019.
- American Institute of Physics. *AAPM Report No. 283. “The Report of Task Group 100 of the AAPM: Application of Risk Analysis Methods to Radiation Therapy Quality Management”*. American Institute of Physics; 2016.
- International Commission on Radiological Protection. Recommendations of the ICRP. ICRP publication 26. *Ann ICRP*. 1977;1(3):3‐6.
- International Commission on Radiological Protection. *Report of Committee II on Permissible Dose for Internal Radiation. ICRP Publication 2*. Pergamon Press; 1960.
- National Council on Radiation Protection and Measurements. *NCRP Report No. 147 – Structural Shielding Design for Medical X‐Ray Imaging Facilities*. National Council on Radiation Protection and Measurements; 2005.
- National Council on Radiation Protection and Measurements. *NCRP Report No. 151 – Structural Shielding Design and Evaluation for Megavoltage X‐ and Gamma‐Ray Radiotherapy Facilities*. National Council on Radiation Protection and Measurements; 2005.
- US Nuclear Regulatory Commission (NRC) Title 10, CFR Part 19–70.
- US DOT Rule 49 CFR Part 300–399, 198.
- Current Federal Regulations, including US Nuclear Regulatory Commission (NRC), US DOT, FDA, Occupational Safety and Health Administration (OSHA), US Public Health Services (PHS), and USEPA

#### 4.1.3 Fundamentals of imaging in medicine

- Bushberg JT, Seibert JA, Leidholt EM, Boone JM. *The Essential Physics of Medical Imaging*. 4th ed. Lippincott Williams and Wilkins; 2020. ISBN 978‐1975103224.
- Samei E, Peck DJ. *Hendee's Physics of Medical Imaging*. 5th ed. Wiley‐Blackwell; 2019. ISBN 978‐0470552209.
- Gonzalez RC, Woods RE. *Digital Image Processing*. 4th ed. Pearson; 2017. ISBN 978‐0133356724.
- Cherry SR, Sorenson JA, Phelps ME. *Physics of Nuclear Medicine*. 4th ed. Elsevier Saunders; 2012. ISBN: 978‐1416051988.
- Bailey DL, Humm JL, Todd‐Pokropek A, van Aswegan A, eds. *Nuclear Medicine Physics: A Handbook for Teachers and Students*. IAEA; 2014. ISBN: 978‐9201438102.

#### 4.1.4 Fundamentals of radiation therapy physics

- Hendee WR, Ibbott GS, Hendee EG. *Radiation Therapy Physics*. 3rd ed. Wiley‐Liss; 2004.
- Johns HE, Cunningham JR. *The Physics of Radiology*. 4th ed. Charles C Thomas; 1983.
- Khan FM. *The Physics of Radiation Therapy*. 4th ed. Lippincott Williams and Wilkins; 2009.
- McDermott PN, Orton CG. *The Physics & Technology of Radiation Therapy*. 2nd ed. Medical Physics Publishing; 2018.
- Podgorsak EB. *Radiation Oncology Physics: A Handbook for Teachers and Students*. IAEA; 2005.
- Rubin P, Bakemeier RF. *Clinical Oncology for Medical Students and Physicians: A Multidisciplinary Approach*. 5th ed. American Cancer Society; 1978.
- Van Dyk J, ed. *The Modern Technology of Radiation Oncology*. Medical Physics Publishing; 1999.
- Van Dyk J, ed. *The Modern Technology of Radiation Oncology*. Vol 2. Medical Physics Publishing; 2005.
- Van Dyk J, ed. *The Modern Technology of Radiation Oncology*. Vol 3. Medical Physics Publishing: 2013.
- Van Dyk J, ed. *The Modern Technology of Radiation Oncology*. Vol 4. Medical Physics Publishing; 2020.

#### 4.1.5 Radiobiology

- Hall EJ, Giaccia AJ. *Radiobiology for the Radiologist*. 8th ed. Lippincott Williams & Wilkins; 2018.
- Joiner MC, van der Kogel AJ. *Basic Clinical Radiobiology*. 5th ed. CRC Press; 2018.
- Mettler FA, Upton AC. *Medical Effects of Ionizing Radiation*. Saunders/Elsevier; 2008.
- National Research Council. *Health Risks from Exposure to Low Levels of Ionizing Radiation: BEIR VII Phase 2*. The National Academies Press; 2006.
- National Council on Radiation Protection and Measurements. *NCRP Report No. 150 – Extrapolation of Radiation‐Induced Cancer Risks from Nonhuman Experimental Systems to Humans*. National Council on Radiation Protection and Measurements; 2005.
- National Council on Radiation Protection and Measurements. *NCRP Report No. 160 – Ionizing Radiation Exposure of the Population of the United States*. National Council on Radiation Protection and Measurements; 2009.
- National Council on Radiation Protection and Measurements. *NCRP Report No. 181 – Evaluation of the Relative Effectiveness of Low‐Energy Photons and Electrons in Inducing Cancer in Humans*. National Council on Radiation Protection and Measurements; 2018.
- National Council on Radiation Protection and Measurements. *NCRP Report No. 184 – Medical Radiation Exposure of Patients in the United States*. National Council on Radiation Protection and Measurements; 2019.
- Weinberg RA. *The Biology of Cancer*. 2nd ed. Garland Science; 2013.

#### 4.1.6 Anatomy and physiology

- Betts JG, DeSaix P, Johnson E, et al. *Anatomy and Physiology*. OpenStax; 2013. ISBN 1938168135.
- Fleckenstein P, Tranum‐Jensen J. *Anatomy in Diagnostic Imaging*. 3rd ed. Wiley‐Blackwell; 2014. ISBN 978‐1‐118‐50048‐4.
- Hanahan D, Weinberg RA. *Hallmarks of Cancer: The Next Generation*. *Cell*. 2011;144:646‐674.
- Nelson DL, Cox MM. *Lehninger Principles of Biochemistry*. 8th ed. Macmillan; 2021. ISBN 9781319230906.
- Marieb EN, Brady PM, Mallatt JB. *Human Anatomy*. 9th ed. Pearson; 2020. ISBN 978‐0135168059.
- Marieb EN, Hoehn KN. *Human Anatomy & Physiology*. 11th ed. Pearson; 2019. ISBN 978‐1292261034.
- Weinberg RA. *The* *Biology of Cancer*. WW Norton; 2013. ISBN 978‐0‐815‐34529‐9.
- Ellis H, Logan BM, Dixon AK, Bowden DJ. *Human Sectional Anatomy*. 4th ed. CRC Press; 2017. ISBN 978‐1498708548.
- Ferrier DR, Champe PC, Harvey RA. *Lippincott Illustrated Reviews: Biochemistry*. 7th ed. Wolters‐Kluwer; 2017. ISBN 9781496344496.
- Alberts B, Johnson A, Lewis J, Morgan D, Raff M, Roberts K, Walter P. *Molecular Biology of the Cell*. 6th ed. Garland Science; 2015. ISBN 978‐0815345244. (Note: 4th edition available on NCBI Bookshelf: https://www.ncbi.nlm.nih.gov/books/NBK21054/)
- Nelson DL, Cox MM, Hoskins AA. *Lehninger Principles of Biochemistry*. 8th ed. Macmillan Learning; 2021. ISBN 978131932238.
- e‐Anatomy. https://doi.org/10.37019/e‐anatomy

#### 4.1.7 Mathematical and statistical methods

- Altman DG. *Practical Statistics for Medical Research*. Chapman & Hall; 1995.
- Arfken GB, Weber HJ. *Mathematical Methods for Physicists*. Academic Press; 1995.
- Armitage P, Berry G. *Statistical Methods in Medical Research*. 3rd ed. Blackwell Scientific Publishing; 1994.
- Bailar JC III, Mosteller F, eds. *Medical Uses of Statistics*. 2nd ed. New England Journal of Medicine; 1992.
- Berry DA, Stangl DK. *Bayesian Biostatistics*. Marcel Dekker; 1996.
- Box GEP, Hunter WG, Hunter JS. *Statistics for Experimenters. An Introduction to Design, Data Analysis, and Model Building*. John Wiley & Sons; 1978.
- Bracewell R. *The Fourier Transform and Its Applications*. 3rd ed. McGraw‐Hill Science/Engineering/Math; 1999.
- Byrne CL. *Signal Processing: A Mathematical Approach*. 2nd ed. CRC Press; 2015.
- Chow S, Liu J. *Design and Analysis of Clinical Trials. Concepts and Methodologies*. John Wiley & Sons; 1998.
- Collett D. *Modelling Survival Data in Medical Research*. Chapman & Hall; 1994.
- Everitt BS, Pickles A*. Statistical Aspects of the Design and Analysis of Clinical Trials*. Imperial College Press; 1999.
- Hastie T, Tibshirani R, Friedman J. *The Elements of Statistical Learning – Data Mining, Inference, and Prediction*. 2nd ed. Springer; 2009.
- James G, Witten D, Hastie T, Tibshirani R. *An Introduction to Statistical Learning: with Applications in R*. Springer; 2013.
- Kochenderfer MJ, Wheeler TA. *Algorithms for Optimization*. The MIT Press; 2019.
- Kreyszig EO. *Introductory Functional Analysis with Applications*. Wiley; 1989.
- McCullagh P, Nelder JA. *Generalized Linear Models*. 2nd ed. Chapman & Hall; 1989.
- Montgomery DC, Peck EA. *Introduction to Linear Regression Analysis*. 2nd ed. John Wiley & Sons; 1992.
- Oppenheim AV, Schafer RW*. Discrete‐Time Signal Processing*. Prentice Hall; 1989.
- Rosner B. *Fundamentals of Biostatistics*. 8th ed. Cengage Learning; 2015.
- Verhulst F. *Nonlinear Differential Equations and Dynamical Systems*. Springer‐Verlag; 1990.

#### 4.1.8 Computational methods and medical informatics

- AAPM. *AAPM Report No. 201. “Quality Management of External Beam Therapy Data Transfer”*. American Association of Physicists in Medicine; 2021.
- Branstetter BF. *Practical Imaging Informatics: Foundations and Applications for PACS Professionals*. Springer; 2010.
- Bushberg JT, Seibert JA, Leidholt EM Jr, Boone JM. *The Essential Physics of Medical Imaging*. 3rd ed. Lippincott Williams & Wilkins; 2012.
- Clark N. *Computer Programming for Beginners: Fundamentals of Programming Terms and Concepts*. CreateSpace Independent Publishing Platform; 2018.
- Ng, Andrew. DeepLearning.AI Programs. https://www.deeplearning.ai/programs/. (Accessed 9/28/2022)
- Manly BFJ. *Randomization, Bootstrap and Monte Carlo Methods in Biology*. 2nd ed. Chapman & Hall; 1997.
- Samei E, Peck DJ. *Hendee's Physics of Medical Imaging*. 5th ed. Wiley‐Blackwell; 2019.
- Starkschall G, Siochi RAC. *Informatics in Radiation Oncology*. CRC Press; 2014.
- Samei E, Pfeiffer DE. *Clinical Imaging Physics: Current and Emerging Practice*. Wiley‐Blackwell; 2020.
- Verhaegen F, Seco J. *Monte Carlo Techniques in Radiation Therapy*. CRC Press; 2016.

#### 4.1.9 Research methods

#### 4.1.10 Professionalism (leadership, ethics, communication)

Leadership

- Medical Physics Leadership Academy website: www.aapm.org/leadership/ (Accessed 9/29/2022)
- Kouzes JM, Posner B. *The Leadership Challenge: How to Make Extraordinary Things Happen in Organization*. 6th ed. Wiley and Sons, Inc.; 2017.
- McCraven WM. *Make Your Bed: Little Things That Can Change Your Life…And Maybe the World*. Grand Central Publishing; 2017.
- Barabás AL. *The Formula: The Universal Laws of Success*. Hachette Book Group, Inc.; 2018.
- Maxwell J. *Developing the Leader Within You 2.0*. Harper Collins; 2018.
- Goleman D. *Working with Emotional Intelligence*. Bantam Books; 2006.
- Bradberry T, Greaves J, Lencioni PM. *Emotional Intelligence 2.0*. TalentSmart; 2009.
- The Arbinger Institute. *The Anatomy of Peace: Resolving the Heart of Conflict*. Berrett‐Koehler Publishers; 2015.
- Patterson K, Grenny J, McMillan R, Switzler A. *Crucial Conversations: Tools for Talking When Stakes are High*. McGraw‐Hill Education; 2002.
- Covey S. *The 7 Habits of Highly Effective People*. Simon & Schuster; 2012.
- Burns JM. *Leadership*. Open Road Media; 2012.
- Adams L. *The Loyalist Team: How Trust, Candor, and Authenticity Create Great Organizations*. PublicAffairs; 2017.
- Goleman D. *Emotional Intelligence: Why It Can Matter More than IQ*. Bloomsbury Publishing; 1996.
- Covey S. *Principle‐Centered Leadership*. Simon & Schuster; 1992.
- Smith P. *Lead with a Story: A Guide to Crafting Business Narratives That Captivate, Convince, and Inspire*. AMACOM; 2012.
- Carnegie D. *How to Win Friends and Influence People*. Pocket Books; 1998.
- Blanchard K, Johnson S. *The One Minute Manager*. William Morrow & Company; 2003.
- Larson CE, LaFasto FMJ. *Teamwork: What Must Go Right/What Can Go Wrong*. Sage Publications; 1989.
- Pfeffer J. *Managing With Power: Politics and Influence in Organizations*. Harvard Business School Press; 1992.

Ethics & professionalism

- American College of Medical Physics, Code of Ethics. http://www.acmp.org/org/code_of_ethics.asp
- American College of Radiology, Code of Ethics. http://www.acr.org/MainMenuCategories/about_us/committees/ethics/code_of_ethics.aspx
- Code of Medical Ethics, American Medical Association. http://www.ama‐assn.org/ama/pub/category/2498.html
- The Nuremberg Code. https://history.nih.gov/research/downloads/nuremberg.pdf
- World Medical Association Declaration of Helsinki – Ethical Principles for Medical Research Involving Human Subjects. https://www.wma.net/policies‐post/wma‐declaration‐of‐helsinki‐ethical‐principles‐for‐medical‐research‐involving‐human‐subjects/
- U.S. Department of Health and Human Services, Office for Human Research Protections. https://www.hhs.gov/ohrp/regulations‐and‐policy/index.html
- U.S. Department of Health and Human Services, Office for Human Research Protections, The Belmont Report. https://www.hhs.gov/ohrp/regulations‐and‐policy/belmont‐report/index.html
- National Institutes of Health. http://www.history.nih.gov/laws%5Chtml%5Cbelmont.htm
- U.S. Department of Health and Human Services, Office for Human Research Protections, Health Information Privacy. https://www.hhs.gov/hipaa/index.html
- Office of Research Integrity. http://ori.dhhs.gov/
- National Education Association code of ethics. http://www.nea.org/aboutnea/code.html
- AAPM/RSNA Onlines Modules on Ethics and Professionalism. https://www.aapm.org/education/webbasedmodules.asp
- Skourou C, Sherouse GW, Bahar N, et al. Code of ethics for the American Association of Physicists in Medicine (Revised): report of Task Group 109. *Med Phys*. 2019;46(4):e79‐e93.
- Serago CF, Burmeister JW, Dunscombe PB, et al. Recommended ethics curriculum for medical physics graduate and residency programs: report of Task Group 159. *Med Phys*. 2010;37(8):4495‐4500.
- Tavani HT. *Ethics & Technology: Ethical Issues in an Age of Information and Communication Technology*. John Wiley & Sons, Inc.; 2007.
- Hogstrom K, Horton J. *Introduction to the Professional Aspects of Medical Physics*. *University of Texas MD Anderson Cancer Center*; 1999.
- Brennan T, Blank L, Cohen J, et al. Medical professionalism in the new millennium: a physician charter. *Ann Intern Med*. 2002;136:243‐246.

Communication

- Penrose AM, Katz SB. *Writing in the Sciences: Exploring Conventions of Scientific Discourse*. The St. Martin's Press; 1998.
- Hogstrom KR, Horton JL. *Introduction to the Professional Aspects of Medical Physics*. The University of Texas M.D. Anderson Cancer Center; 1999.
- Epstein RM, Street RL. *Patient‐Centered Communication in Cancer Care*. National Cancer Institute, NIH Publication No. 07‐6225; 2007.

### 4.2 Additional topics (optional)

#### 4.2.1 Biology/oncology/medicine

#### 4.2.2 Advanced physics, engineering, computer science

#### 4.2.3 Frontiers in medical physics and opportunities outside of medical physics

#### 4.2.4 Business aspects of medical physics

#### 4.2.5 Teaching for medical physicists

- Starkschall G. Editorial: Medical physicists as educators. *J Appl Clin Med Phys*. 2009;10(3):1‐2.
- Mazur E. *Peer Instruction: A User's Manual*. Prentice‐Hall Inc.; 1997.
- Redish EF. *Teaching Physics: with the Physics Suite*. John Wiley & Sons; 2004. Available free online at http://www2.physics.umd.edu/~redish/Book/
- Defined Learning. What is Project‐Based Learning? website; 2019. https://www.definedstem.com/blog/what‐is‐project‐based‐learning/
- Buck Institute for Education. What is PBL? website; 2019. https://www.pblworks.org/what‐is‐pbl
- Kry S. Best practices: flipped learning in the medical physics classroom. Presentation from the 2018 AAPM Workshop on Improving the Teaching and Mentoring of Medical Physics. AAPM Virtual Library. Available on the AAPM website at: https://www.aapm.org/education/VL/vl.asp?id=12667
- Howell R. Best practices: project‐based learning (PBL) in medical physics. Presentation from the 2018 AAPM Workshop on Improving the Teaching and Mentoring of Medical Physics. AAPM Virtual Library. Available on the AAPM website at: https://www.aapm.org/education/VL/vl.asp?id=12661
- Knowles M. *The Adult Learner: A Neglected Species*. 3rd ed. Gulf Publishing; 1984.
- Webster RS. In defence of the lecture. *Aust J Teach Educ*. 2015;40(10). Available online at https://files.eric.ed.gov/fulltext/EJ1078748.pdf.
- Armstrong P. Bloom's Taxonomy. website; 2021. https://cft.vanderbilt.edu/guides‐sub‐pages/blooms‐taxonomy/
- National Research Council. *How People Learn: Brain, Mind, Experience, and School*. National Academy Press; 2000.

### 4.3 Diagnostic imaging specialization (optional)

- Bushberg JT, Seibert JA, Leidholt EM, Boone JM. *The Essential Physics of Medical Imaging*. 4th ed. Lippincott Williams and Wilkins; 2020. ISBN 978‐1975103224.
- Samei E, Peck DJ. *Hendee's Physics of Medical Imaging*. 5th ed. Wiley‐Blackwell; 2019. ISBN 978‐0470552209.
- Prince JL, Links JM. *Medical Imaging Signals and Systems*. 2nd ed. Pearson; 2014. ISBN 978‐0132145183.
- Epstein CL. *Introduction to the Mathematics of Medical Imaging*. 2nd ed. SIAM; 2008. ISBN 978‐0898716429.
- Barrett HH, Meyers KJ. *Foundations of Image Science*. John Wiley & Sons; 2004. ISBN 978‐0471153009.
- Deans SR. *The Radon Transform and Some of Its Applications*. Dover Publications; 2007. ISBN 978‐0486462417.
- Jain AK. *Fundamentals of Digital Image Processing*. Pearson; 1988. ISBN 978‐0133361650.
- Gonzalez RC, Woods RE. *Digital Image Processing*. 4th ed. Pearson; 2017. ISBN 978‐0133356724.
- Knoll GF. *Radiation Detection and Measurement*. 4th ed. John Wiley & Sons; 2010. ISBN 978‐0470131480.
- Hsieh J. *Computed Tomography: Principles, Design, Artifacts, and Recent Advances*. 3rd ed. SPIE Press; 2015. ISBN 978‐1628418255.
- Bushong SC, Clarke G. *Magnetic Resonance Imaging: Physical and Biological Principles*. 4th ed. Mosby; 2014. ISBN 978‐0323073547.
- Nishimura D. *Principles of Magnetic Resonance Imaging*. Lulu; 2010.
- Brown RW, Chieng Y‐CN, Haacke EM, Thompson MR, Venkatesan R. *Magnetic Resonance Imaging: Physical Principles and Sequence Design*. 2nd ed. Wiley Blackwell; 2014. ISBN 978‐0471720850.
- National Council on Radiation Protection and Measurements. *NCRP Report No. 147. “Structural Shielding Design for Medical X‐ray Imaging Facilities”*. National Council on Radiation Protection and Measurements; 2004. ISBN 0929600835.

### 4.4 Nuclear medicine specialization (optional)

- Cherry SR, Sorenson JA, Phelps ME. *Physics of Nuclear Medicine*. 4th ed. Elsevier Saunders; 2012. ISBN: 978‐1416051988.
- Bailey DL, Humm JL, Todd‐Pokropek A, van Aswegan A, eds. *Nuclear Medicine Physics: A Handbook for Teachers and Students*. IAEA; 2014. ISBN: 978‐9201438102.
- Loevinger R, Budinger TF, Watson EE. *MIRD Primer for Absorbed Dose Calculations*. Society of Nuclear Medicine; 1991. ISBN: 0‐932004385.
- Sgouros G, Bodei L, McDevitt M, Nedrow JR. Radiopharmaceutical therapy in cancer: clinical advances and challenges. *Nat Rev Drug Discovery*. 2020;19(9):589‐608.
- Madsen MT, Anderson JA, Halama JR, et al. AAPM Task Group 108: PET and PET/CT shielding requirements. *Med Phys*. 2006;33(1):4‐15.
- Knoll GF. *Radiation Detection and Measurement*. 4th ed. Wiley; 2010. ISBN: 978‐0470131480.
- Volterrani D, Erba PA, Carrió I, Strauss HW, Mariani G. *Nuclear Medicine Textbook*. Springer; 2019. ISBN: 978‐3‐319‐95564‐3.
- Sandler MP, Coleman RE, Patton JA, Wackers FJTh, Gottschalk A. *Diagnostic Nuclear Medicine*. 4th ed. Lippincott, Williams & Williams; 2003. ISBN: 978‐0781732529.
- IAEA. *Nuclear Medicine Resources Manual 2020 Edition, Human Health Series No. 37*. IAEA; 2020.

### 4.5 Radiation therapy specialization (optional)

- AAPM. *AAPM Report No. 17. “The Physical Aspects of Total and Half Body Photon Irradiation”*. American Institute of Physics; 1986.
- AAPM. *AAPM Report No. 23. “Total Skin Electron Therapy: Technique and Dosimetry”*. American Institute of Physics; 1987.
- AAPM. *AAPM Report No. 46. “Comprehensive QA for Radiation Oncology”*. American Institute of Physics; 1994.
- AAPM. *AAPM Report No. 51. “Dosimetry of Interstitial Brachytherapy Sources”*. American Institute of Physics; 1995.
- AAPM. *AAPM Report No. 54. “Stereotactic Radiosurgery”*. American Institute of Physics; 1995.
- AAPM. *AAPM Report No. 59. “Code of Practice for Brachytherapy Physics: Report of the AAPM Radiation Therapy Committee Task Group No. 56”*. American Institute of Physics; 1997.
- AAPM. *AAPM Report No. 62. “American Association of Physicists in Medicine Radiation Therapy Committee Task Group 53: Quality Assurance for Clinical Radiotherapy Treatment Planning”*. American Institute of Physics; 1998.
- AAPM. *AAPM Report No. 67. “AAPM's TG‐51 protocol for clinical reference dosimetry of high‐energy photon and electron beams”*. American Institute of Physics; 1999.
- AAPM. *AAPM Report No. 76. “AAPM Protocol for 40–300 kV X‐Ray Beam Dosimetry in Radiotherapy and Radiobiology”*. American Institute of Physics; 2001.
- AAPM. *AAPM Report No. 91. “The Management of Respiratory Motion in Radiation Oncology”*. American Institute of Physics; 2006.
- AAPM. *AAPM Report No. 99. “Recommendations for Clinical Electron Beam Dosimetry: Supplement to the Recommendations of Task Group 25”*. American Institute of Physics; 2009.
- AAPM. *AAPM Report No. 100. “Acceptance Testing and Quality Assurance Procedures for Magnetic Resonance Imaging Facilities”*. American Institute of Physics; 2010.
- AAPM. *AAPM Report No. 101. “Stereotactic Body Radiation Therapy: The Report of AAPM Task Group 101”*. American Institute of Physics; 2010.
- AAPM. *AAPM Report No. 106. “Accelerator Beam Data Commissioning Equipment and Procedures: Report of the TG‐106 of the Therapy Physics Committee of the AAPM”*. American Institute of Physics; 2008.
- AAPM. *AAPM Report No. 119. “IMRT Commissioning: Multiple Institution Planning and Dosimetry Comparisons, a Report from AAPM Task Group 119”*. American Institute of Physics; 2009.
- AAPM. *AAPM Report No. 142. “Task Group 142 Report: Quality Assurance of Medical Accelerators”*. American Institute of Physics; 2009.
- AAPM. *AAPM Report No. 148. “QA for Helical Tomotherapy: Report of the AAPM Task Group 148”*. American Institute of Physics; 2010.
- AAPM. *AAPM Report No. 218. “Tolerance Limits and Methodologies for IMRT Measurement‐Based Verification QA: Recommendations of AAPM Task Group No. 218”*. American Institute of Physics; 2018.
- AAPM. *AAPM Report No. 263. “Standardizing Nomenclatures in Radiation Oncology”*. American Institute of Physics; 2018.
- AAPM. *AAPM Report No. 275. “Strategies for Effective Physics Plan and Chart Review in Radiation Therapy: Report of AAPM Task Group 275”*. American Institute of Physics; 2020.
- AAPM. *AAPM Report No. 283. “The report of Task Group 100 of the AAPM: Application of Risk Analysis Methods to Radiation Therapy Quality Management”*. American Institute of Physics; 2016.
- AAPM Monographs and Proceedings. https://medicalphysics.org/SimpleCMS.php?content=booklist.php&category=monographs
- National Council on Radiation Protection and Measurements. *NCRP Report No. 147 – Structural Shielding Design for Medical X‐Ray Imaging Facilities*. National Council on Radiation Protection and Measurements; 2005.
- National Council on Radiation Protection and Measurements. *NCRP Report No. 151 – Structural Shielding Design and Evaluation for Megavoltage X‐ and Gamma‐Ray Radiotherapy Facilities*. National Council on Radiation Protection and Measurements; 2005.
- Bentel GC, Nelson CE, Noell KT. *Treatment Planning & Dose Calculation in Radiation Oncology*. 4th ed. Pergamon Press; 1989.
- Greene D, Williams PC. *Linear Accelerators for Radiation Therapy*. 2nd ed. Taylor & Francis; 1997.
- Pawlicki T, Scanderberg DJ, Starkschall G. *Hendee's Radiation Therapy Physics*. 4th ed. John Wiley & Sons; 2015.
- International Commission on Radiation Units and Measurements. *ICRU Report No. 33. “Radiation Quantities & Units”*. International Commission on Radiation Units and Measurements; 1980.
- ICRU. *ICRU Report No. 50. “Prescribing, Recording and Reporting Photon Beam Therapy”*. Oxford University Press; 1993.
- ICRU. *ICRU Report No. 62. “Prescribing, Recording and Reporting Photon Beam Therapy (Supplement to ICRU 50)”*. Oxford University Press; 1999.
- ICRU. *ICRU Report No. 71. “Prescribing, Recording and Reporting Electron Beam Therapy (Supplement to ICRU 50)”*. Oxford University Press; 2004.
- ICRU. *ICRU Report No. 78. “Prescribing, Recording and Reporting Proton Beam Therapy”*. Oxford University Press; 2007.
- ICRU. *ICRU Report No. 83. “Prescribing, Recording and Reporting Intensity Modulated Photon Beam Therapy (IMRT)”*. Oxford University Press; 2010.
- ICRU. *ICRU Report No. 89. “ICRU Report 89, Prescribing, Recording, and Reporting Brachytherapy for Cancer of the Cervix”*. Oxford University Press; 2016.
- Johns HE, Cunningham JR. *The Physics of Radiology*. 4th ed. Charles C Thomas; 1983.
- Karzmark CJ, Nunan CS. *Medical Electron Accelerators*. McGraw‐Hill; 1993.
- Gibbons JP. *Khan's The Physics of Radiation Therapy*. 6th ed. Walters Kluwer; 2020.
- Klevenhagen SC. *Physics and Dosimetry of Therapy Electron Beams*. Medical Physics Publishing; 1993.
- McDermott PN, Orton CG. *The Physics & Technology of Radiation Therapy*. 2nd ed. Medical Physics Publishing; 2018.
- McGinley P. *Shielding Techniques for Radiation Oncology Facilities*. 2nd ed. Medical Physics Publishing; 2002.
- Metcalfe P, Kron T, Hoban P. *The Physics of Radiotherapy X‐Rays and Electrons*. Medical Physics Publishing; 2007.
- Podgorsak EB. *Radiation Oncology Physics: A Handbook for Teachers and Students*. IAEA; 2005.
- Rubin P, Bakemeier RF. *Clinical Oncology for Medical Students and Physicians: A Multidisciplinary Approach*. 5th ed. American Cancer Society; 1978.
- Van Dyk J, ed. *The Modern Technology of Radiation Oncology*. Medical Physics Publishing; 1999.
- Van Dyk J, ed. *The Modern Technology of Radiation Oncology*. Vol 2. Medical Physics Publishing; 2005.
- Van Dyk J, ed. *The Modern Technology of Radiation Oncology*. Vol 3. Medical Physics Publishing; 2013.
- Van Dyk J, ed. *The Modern Technology of Radiation Oncology*. Vol 4. Medical Physics Publishing; 2020.
- Khan F, Gibbons JP, Sperduto PW. *Treatment Planning in Radiation Oncology*. Wolters Kluwer; 2016.
- Benedict SH, Schlesinger DJ, Goetsch SJ, Kavanagh BD. *Stereotactic Radiosurgery and Stereotactic Body Radiation Therapy*. CRC Press; 2015.
- Webb S. *Intensity‐Modulated Radiation Therapy*. Institute of Physics Publishing; 2001.
- Devlin PM, Cormack RA, Holloway CL, Stewart AJ. *Brachytherapy: Applications and Techniques*. Demos Medical; 2016.

### 4.6 Medical health physics specialization (optional)

- Madsen MT, Anderson JA, Halama JR, et al. AAPM Task Group 108: PET and PET/CT shielding requirements. *Med Phys*. 2006;33(1):4‐15.
- Bushberg JT, Seibert JA, Leidholt EM Jr, Boone JM. *The Essential Physics of Medical Imaging*. 4th ed. Lippincott William and Wilkins; 2020.
- Johnson TE. *Introduction to Health Physics*. 5th ed. McGraw‐Hill; 2017.
- Knoll GF. *Radiation Detection and Measurement*. 4th ed. Wiley; 2011.
- Mossman K. *Radiation Risks in Perspective*. CRC Press; 2007.
- National Council on Radiation Protection and Measurements. *NCRP Report No. 147 – Structural Shielding Design for Medical X‐Ray Imaging Facilities*. National Council on Radiation Protection and Measurements; 2005.
- National Council on Radiation Protection and Measurements. *NCRP Report No. 151 – Structural Shielding Design and Evaluation for Megavoltage X‐ and Gamma‐Ray Radiotherapy Facilities*. National Council on Radiation Protection and Measurements; 2005.
- National Research Council. *Health Risks from Exposure to Low Levels of Ionizing Radiation: BEIR VII Phase 2*. The National Academies Press; 2006.
- National Research Council. *Health Effects of Exposure to Radon: BEIR VI*. The National Academies Press; 1996.
- Stabin M. *Radiation Protection and Dosimetry: An Introduction to Health Physics*. Springer; 2007.
- Stabin M. *Fundamentals of Nuclear Medicine Dosimetry*. Springer; 2008.
- http://nucleardata.nuclear.lu.se/toi/
- http://www.doseinfo‐radar.com/
- https://www.nrc.gov/about‐nrc/emerg‐preparedness.html
- https://www.nist.gov/pml/radionuclide‐half‐life‐measurements

### 4.7 Industry specialization (optional)

- http://fda.yorkcast.com/webcast/Play/d91af554691c4260b5eca0b2a28e636b1d
- http://www.fda.gov/downloads/MedicalDevices/DeviceRegulationandGuidance/GuidanceDocuments/UCM284443.pdf
- https://www.fda.gov/media/82395/download
- https://www.accessdata.fda.gov/scripts/cdrh/cfdocs/cfpcd/classification.cfm?id=5376
- Mason DJ, Perez A, McLemore MR, Dickson E. *Policy & Politics in Nursing and Health Care*. 8th ed. Saunders; 2020. https://www.brookings.edu/project/usc‐brookings‐schaeffer‐initiative‐for‐health‐policy/
- https://www.brookings.edu/series/health‐policy‐101/
- https://www.ahip.org/
- https://www.aaas.org/resources/
- Bardach E, Patashnik EM. *A Practical Guide for Policy Analysis*. 5th ed. SAGE Publishing; 2016.
- https://www.iaea.org/about/policy
- https://www.who.int/topics/health_policy/en/

## AUTHOR CONTRIBUTIONS

All authors meet the following criteria:

- substantial contributions to the conception or design of the work; or the acquisition, analysis, or interpretation of data for the work;
- drafting the work or revising it critically for important intellectual content;
- final approval of the version to be published;
- agreement to be accountable for all aspects of the work in ensuring that questions related to the accuracy or integrity of any part of the work are appropriately investigated and resolved.

## CONFLICT OF INTEREST

The authors declare no conflicts of interest.

## References

[1] AAPM . AAPM Report No. 44. Academic Program for Master of Science Degree in Medical Physics. American Institute of Physics; 1993.

[2] AAPM . AAPM Report No. 79. Academic Program Recommendations for Graduate Degrees in Medical Physics. American Institute of Physics; 2002.

[3] AAPM . AAPM Report No. 197. Academic Program Recommendations for Graduate Degrees in Medical Physics. American Institute of Physics; 2009.

[4] AAPM . AAPM Report No. 197S. The Essential Medical Physics Didactic Elements for Physicists Entering the Profession through an Alternative Pathway: A Recommendation from the AAPM Working Group on the Revision of Reports 44 & 79. American Institute of Physics; 2011.

[5] AAPM . AAPM Report No. 298. Certificate Program /Alternative Pathway Candidate Education and Training: Recommendations of AAPM Task Group No. 298. American Institute of Physics (In Press).

[6] AAPM . AAPM Report No. 249. Essentials and Guidelines for Clinical Medical Physics Residency Training Programs. American Institute of Physics; 2002. 10.1120/jacmp.v15i3.4763 PMC5711071 24892354

[7] Burmeister JW , Coffey CW , Hazle JD , et al. AAPM report no. 373. The content, structure, and value of the professional doctorate in medical physics (DMP). J Appl Clin Med Phys. 2022:e13771. 36107002 10.1002/acm2.13771 PMC9588257

[8] Bloom BS , Engelhart MD , Furst EJ , Hill WH , Krathwohl DR . Taxonomy of Educational Objectives: The Classification of Educational Goals. Handbook I: Cognitive Domain. David McKay Company; 1956.

