# Erratum: “Academic program recommendations for graduate degrees in medical physics: AAPM Report No. 365 (Revision of Report No. 197)”

**DOI:** 10.1002/acm2.13887

**Published:** 2023-02-16

The subsections listed in Sections 3.1.5–3.1.10 and 3.5 were misnumbered. The online version of this article has been corrected accordingly. The typesetters apologize for this error.

